# Frequently asked questions about in vivo chlorophyll fluorescence: practical issues

**DOI:** 10.1007/s11120-014-0024-6

**Published:** 2014-08-15

**Authors:** Hazem M. Kalaji, Gert Schansker, Richard J. Ladle, Vasilij Goltsev, Karolina Bosa, Suleyman I. Allakhverdiev, Marian Brestic, Filippo Bussotti, Angeles Calatayud, Piotr Dąbrowski, Nabil I. Elsheery, Lorenzo Ferroni, Lucia Guidi, Sander W. Hogewoning, Anjana Jajoo, Amarendra N. Misra, Sergio G. Nebauer, Simonetta Pancaldi, Consuelo Penella, DorothyBelle Poli, Martina Pollastrini, Zdzislawa B. Romanowska-Duda, Beata Rutkowska, João Serôdio, Kancherla Suresh, Wiesław Szulc, Eduardo Tambussi, Marcos Yanniccari, Marek Zivcak

**Affiliations:** 1 Department of Plant Physiology, Faculty of Agriculture and Biology, Warsaw University of Life Sciences – SGGW, Nowoursynowska 159, 02-776 Warsaw, Poland; 2 Avenue des Amazones 2, 1226 Chêne-Bougeries, Switzerland; 3 Institute of Biological and Health Sciences, Federal University of Alagoas, Praça Afrânio Jorge, s/n, Prado, Maceió, AL Brazil; 4 Department of Biophysics and Radiobiology, Faculty of Biology, St. Kliment Ohridski University of Sofia, 8 Dr. Tzankov Blvd., 1164 Sofia, Bulgaria; 5 Department of Pomology, Faculty of Horticulture, Biotechnology and Landscape Architecture, Warsaw University of Life Sciences – SGGW, Nowoursynowska 159, 02-776 Warsaw, Poland; 6 Institute of Plant Physiology, Russian Academy of Sciences, Botanicheskaya Street 35, Moscow, 127276 Russia; 7 Institute of Basic Biological Problems, Russian Academy of Sciences, Pushchino, Moscow Region, 142290 Russia; 8 Department of Plant Physiology, Slovak Agricultural University, Tr. A. Hlinku 2, 949 76 Nitra, Slovak Republic; 9 Department of Agri-Food Production and Environmental Science (DISPAA), University of Florence, Piazzale delle Cascine 28, 50144 Florence, Italy; 10 Departamento de Horticultura, Instituto Valenciano de Investigaciones Agrarias, Ctra. Moncada-Náquera Km 4.5, Moncada, 46113 Valencia, Spain; 11 Department of Environmental Improvement, Faculty of Civil and Environmental Engineering, Warsaw University of Life Sciences – SGGW, Nowoursynowska 159, 02-776 Warsaw, Poland; 12 Agricultural Botany Department, Faculty of Agriculture, Tanta University, Tanta, Egypt; 13 Department of Life Sciences and Biotechnologies, University of Ferrara, Corso Ercole I d’Este 32, 44121 Ferrara, Italy; 14 Department of Agriculture, Food and Environment, Via del Borghetto, 80, 56124 Pisa, Italy; 15 Plant Lighting BV, Veilingweg 46, 3981 PC Bunnik, The Netherlands; 16 School of Life Sciences, Devi Ahilya University, Indore, 452 001 M.P India; 17 Centre for Life Sciences, Central University of Jharkhand, Ratu-Lohardaga Road, Ranchi, 835205 India; 18 Departamento de Producción vegetal, Universitat Politècnica de València, C de Vera sn, 46022 Valencia, Spain; 19 Department of Biology, Roanoke College, 221 College Lane, Salem, VA 24153 USA; 20 Department of Ecophysiology and Plant Development, University of Lodz, Banacha 12/16, Lodz, 90-237 Poland; 21 Agricultural Chemistry Department, Faculty of Agriculture and Biology, Warsaw University of Life Sciences – SGGW, Nowoursynowska 159, 02-776 Warsaw, Poland; 22 Departamento de Biologia, CESAM – Centro de Estudos do Ambiente e do Mar, Universidade de Aveiro, Campus de Santiago, 3810-193 Aveiro, Portugal; 23 Directorate of Oil Palm Research, West Godavari Dt., Pedavegi, 534 450 Andhra Pradesh India; 24 Institute of Plant Physiology, INFIVE (Universidad Nacional de La Plata – Consejo Nacional de Investigaciones Científicas y Técnicas), Diagonal 113 N°495, 327 La Plata, Argentina

**Keywords:** Chlorophyll *a* fluorescence, Fluorescence imaging, Complementary techniques, Frequently asked questions, Plant stress monitoring, Photosynthesis

## Abstract

The aim of this educational review is to provide practical information on the hardware, methodology, and the hands on application of chlorophyll (Chl) *a* fluorescence technology. We present the paper in a question and answer format like frequently asked questions. Although nearly all information on the application of Chl *a* fluorescence can be found in the literature, it is not always easily accessible. This paper is primarily aimed at scientists who have some experience with the application of Chl *a* fluorescence but are still in the process of discovering what it all means and how it can be used. Topics discussed are (among other things) the kind of information that can be obtained using different fluorescence techniques, the interpretation of Chl *a* fluorescence signals, specific applications of these techniques, and practical advice on different subjects, such as on the length of dark adaptation before measurement of the Chl *a* fluorescence transient. The paper also provides the physiological background for some of the applied procedures. It also serves as a source of reference for experienced scientists.

## Introduction

The measurement of chlorophyll (Chl) *a* fluorescence is one of the most widely used methods to probe photosynthesis (see Papageorgiou and Govindjee 2004 for reviews on application of Chl *a* fluorescence to different aspects of photosynthesis; also see Govindjee (2004) for an overview of important publications on Chl *a* fluorescence). Any researcher who tries to find his or her way in the fluorescence literature will initially be overwhelmed by the number of published articles and by all the conflicting ideas. Such a researcher will also quickly discover that it is not easy to find an answer for many simple and basic questions. We plan to fill this gap in this educational review focusing mainly on plants, green algae, and diatoms.

The Chl *a* fluorescence signal is very rich in its content; it is very sensitive to changes in photosynthesis and can be recorded with great precision. Many processes affect the fluorescence yield and/or intensity, and using a variety of light protocols (flashes, pulses, continuous light, etc.), different processes can be studied. However, most authors have used only a limited set of experimental protocols based on methods that have been developed over time.

With the available commercial equipment, it is very easy to make a fluorescence measurement, but as the literature shows, the interpretation of such measurements is still very contentious. There is not even agreement on the processes that determine the fluorescence rise from *F*_O_ to *F*_M_, i.e., the variable fluorescence (*F*_V_). The dominant interpretation assumes that the variable fluorescence is determined by the redox state of *Q*_A_, the first quinone acceptor of PSII, as originally proposed by Duysens and Sweers (1963) and recently defended by Stirbet and Govindjee (2012). Delosme (1967) on the other hand argued that *Q*_A_ was not enough and that there was another important process explaining part of *F*_V_. This position has recently been supported and extended by Schansker et al. (2011, 2014); see Question 21 for a broader discussion of this point.

Another attractive feature of Chl *a* fluorescence is its non-invasive character, which allows the measurement on leaves and even on canopies of trees during long periods of time. A range of instruments has been developed focusing on different aspects of photosynthesis and on different properties of Chl *a* fluorescence. An overview will be given here of the available types of instruments, and we will discuss also what kind of information can be obtained with these instruments.

It is important to understand that a fluorescence value by itself has no meaning. A well-defined reference state for the photosynthetic sample measured is needed to allow an appropriate interpretation of the data. Processes that relax following illumination will be discussed here as well as the time needed to reach the dark-adapted state, which is an important reference state.

A widely read introductory paper on the use of Chl *a* fluorescence is by Maxwell and Johnson (2000), and two more recent papers treating the application of Chl *a* fluorescence techniques are by Logan et al. (2007) and Murchie and Lawson (2013). These papers focus on the analysis of what is called the steady state: the stable photosynthetic activity after 5–10 min of illumination at a chosen light intensity. Here, our focus is broader, considering a wider range of fluorescence techniques. We make the point that interpretation of fluorescence data can be improved making use, at the same time, of different classes of fluorescence techniques, as well as by the use of complementary techniques such as gas exchange and 820 nm transmission/absorption measurements. We also emphasize that there are still controversies with respect to the interpretation of Chl *a* fluorescence data.

The educational review is meant to be a starting point for researchers interested in further exploiting Chl *a* fluorescence measurements to understand photosynthetic systems. Some questions arise are trivial, e.g.,

## Question 1: should the instrument be called fluorimeter or fluorometer?

Both versions are allowed, the former being British-English and the latter American-English.

Answers to other questions may make the difference between a successful and a failed experiment.

## Question 2. Which types of instruments are available for fluorescence measurements?

**Fig. 1:**
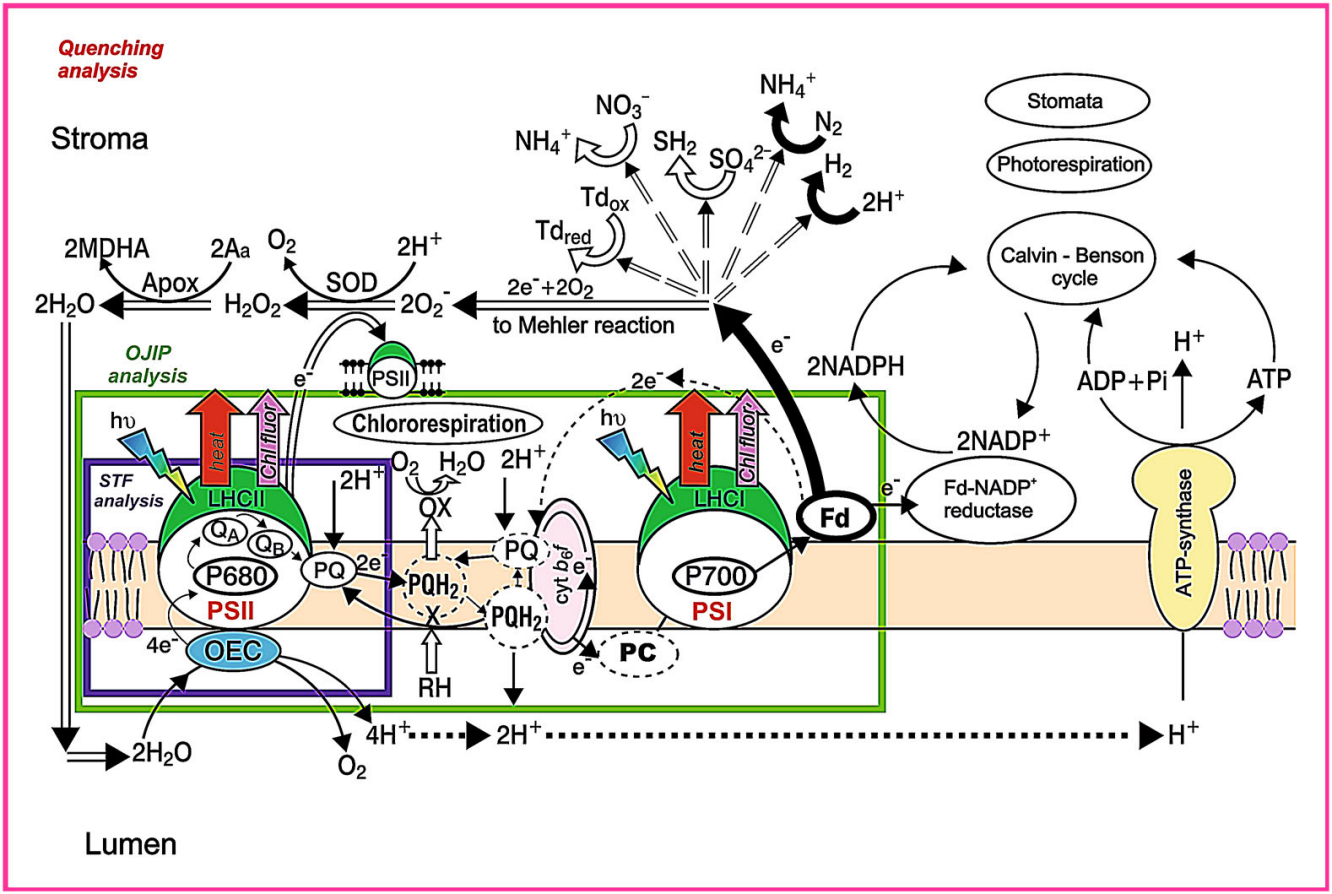
The processes that can be studied analyzing the fluorescence decay following a single turnover flash, the analysis of OJIP transients, or the quenching analysis. With the analysis of the fluorescence decay kinetics (STF analysis, *purple line*), it is possible to obtain information on electron transport reactions inside PSII and via the occupancy state of the *Q*_B_-site on the PQ-pool redox state; OJIP transients (*green line*) can be used to obtain information on the redox state of the photosynthetic ETC, on the stoichiometry of the components of the ETC and on the relative PSII antenna size; the quenching analysis (*rosa line*) gives information on dynamic processes, electron flow, under steady state conditions, is sensitive to short-term regulatory processes in the antenna (see text) and to Calvin–Benson cycle activity, changes in photorespiration and stomatal opening (modified from Kalaji and Loboda 2010)

For a rough classification of fluorescence instruments used to probe electron transfer reactions involving photosystem II (PSII) and/or photosystem I (PSI), three major classes can be distinguished (see Fig. 1 for an illustration of this classification and see Question 33 for a discussion of fast repetition rate (FRR) measurements and equipment).

1. Instruments based on short light flashes (few μs or less). With such instruments, information on the electron transfer reactions within PSII can be obtained: re-oxidation kinetics of *Q*_A_^−^ via forward electron transfer to *Q*_B_ or recombination with the donor side of PSII (see Fig. 2).

**Fig. 2:**
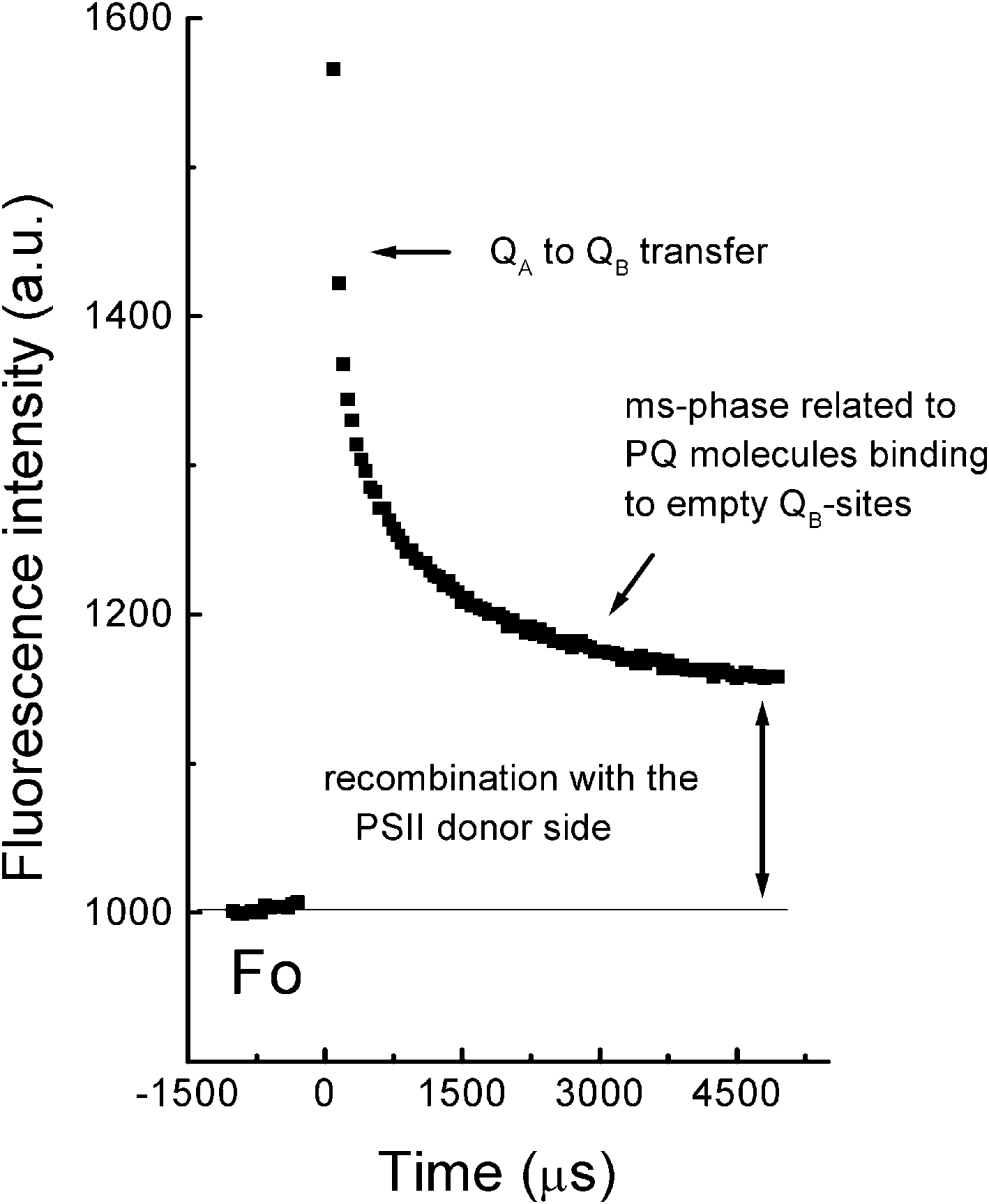
Example of the fluorescence decay kinetics following a single turnover xenon flash to a suspension of PSII-enriched membranes isolated from spinach. Several pre-flashes had been given to induce a partial reduction of the PQ-pool (G. Schansker, unpublished data)
2. Instruments based on a saturating pulse (few hundred ms strong light). With such instruments, information on the photosynthetic electron transport chain (ETC) can be obtained: reduction kinetics of the ETC, PSII antenna size, relative content of ETC components like PSI (see Fig. 3).

**Fig. 3:**
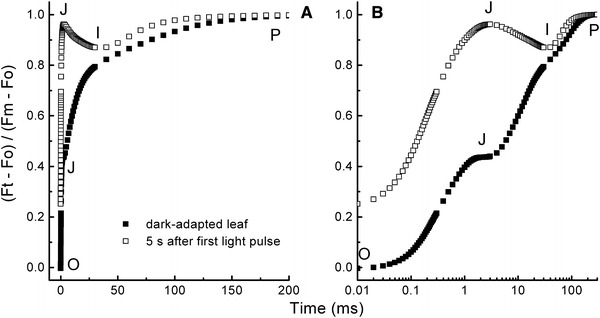
OJIP transients (double normalized between O and P) measured on a bean leaf (*Phaseolus vulgaris*) shown on a linear timescale (**a**) and a logarithmic timescale (**b**). A measurement on dark adapted (*closed symbols*) which has an oxidized PQ-pool and a low J-step and a measurement made 5 s later (*open symbols*) where *Q*_A_ had become re-oxidized in part of the PSII RCs due to recombination (O level considerably below P), the PQ-pool is still almost completely reduced (J level near P), and the acceptor side of PSI is almost completely re-oxidized (I level close to that of the dark-adapted state) (G. Schansker, unpublished data)
3. Instruments designed to study the steady state (relatively stable photosynthetic activity after 5–10 min of illumination). With such instruments, light-induced regulatory mechanisms, interaction between ETC, Calvin–Benson cycle, stomatal opening, and photorespiration (the process initiated when the enzyme Rubisco reacts with O_2_ instead of CO_2_) are studied (see Fig. 4).

**Fig. 4:**
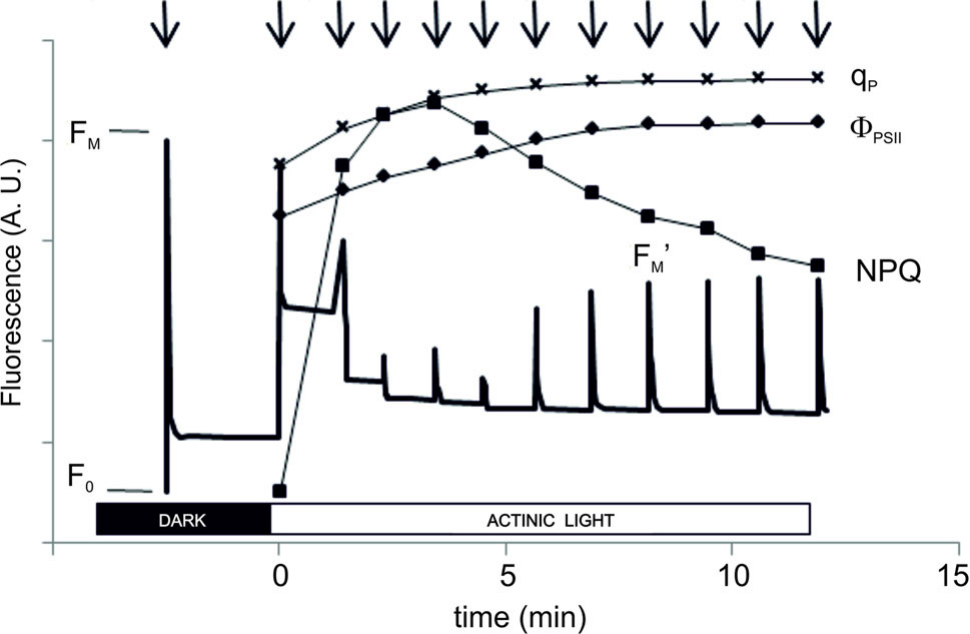
Slow Chlorophyll *a* fluorescence kinetics (in arbitrary units) using a PAM-2100 fluorometer. The dark-adapted leaf is illuminated with weak modulated measuring light to give the zero fluorescence level *F*_0_. Application of a saturation pulse (SP) allows measurement of the maximum fluorescence level in the dark *F*_M_. Photosynthesis is then activated by an actinic light source (in this case 250 μmol photons m^−2^ s^−1^). SPs during the light phase were triggered spaced 1 min apart (indicated by *arrows*) to determine the maximum fluorescence intensity in the light (*F*_M_′), and for each SP, q_P_, *Φ*_PSII,_ and NPQ parameters were calculated, and these are indicated in the figure (Penella et al. unpublished data)

### Flash fluorescence measurements

Figure 2 shows an example of a typical flash fluorescence experiment. These measurements are based on the concept of a single turnover flash (STF). An STF has to meet two requirements: (1) The intensity of a STF must be high enough to excite the antennae of all PSII reaction centers (RCs) followed by a charge separation in all PSII RCs leading to a reduction of essentially all *Q*_A_; (2) A STF must be short enough to induce only one charge separation in each PSII RC. In practice, this situation is never completely reached, and either misses or double hits are induced in a small fraction of PSII RCs (see e.g., Kok et al. 1970; Shinkarev 2005). The re-oxidation of *Q*_A_^−^ can then be followed: in active RCs, most electrons will be transferred to *Q*_B_ and following a second flash to *Q*_B_^−^ (see Fig. 2). The first reaction has a half-time of 100–200 μs, and the second reaction has a half-time of 400–600 μs (reviewed by Petrouleas and Crofts 2005). If no PQ is bound to the *Q*_B_-site, the electron on *Q*_A_^−^ has to wait, till a PQ molecule binds to the *Q*_B_-site, and this process can take a few ms (Crofts and Wraight 1983). In the case of inactive PSII centers, forward electron transfer cannot take place, and re-oxidation of *Q*_*A*_− occurs via a recombination reaction with the donor side of PSII (Lavergne 1982a; Chylla et al. 1987; Lavergne and Leci 1993; Schansker and Strasser 2005). These instruments can also be used to study the S-states (oxidation states S0, S1, S2, S3 and S4) of the oxygen evolving complex of PSII. A series of STFs induces period-4 oscillations in the *F*_O_-level as a function of the S-states (see Delosme 1972; Delrieu 1998; Ioannidis et al. 2000 for examples of such measurements).

To probe the oxidation of reduced *Q*_A_ following a saturating flash, there are two possible approaches:

1. The easiest method makes use of low-intensity modulated light, which excites only a small fraction of the PSII RCs per unit of time. Figure 2 shows an example of such a measurement. For control samples, in which re-oxidation of *Q*_A_^−^ via forward electron transport can occur, this approach works well. However, when the sample is inhibited, e.g., by an electron transfer inhibitor such as DCMU (3-(3,4-dichlorophenyl)-1,1-dimethylurea), which displaces *Q*_B_ from its binding site (Velthuys 1981; Lavergne 1982b), the low-intensity modulated light leads to the accumulation of a considerable population of *Q*_A_^−^ complicating the analysis of the experiment, because re-oxidation of *Q*_A_^−^ by recombination with the donor side is much slower than forward electron transport to *Q*_B_.
2. The second method uses a combination of a STF followed by a probe flash that probes the redox state of *Q*_A_ at the time of the probe flash (this is called a pump–probe experiment) (Mauzerall 1972; Robinson and Crofts 1983). The intensity of the probe flash is much lower than that of the STF. In this case, the experiment is repeated many times and each time at a variable time t after the STF, a probe flash is given to probe the redox state of *Q*_A_. In this way, the re-oxidation kinetics are constructed point by point. The actinic light problem, described above for DCMU inhibited samples, does not exist in this case. On the other hand, identical samples do not exist, and therefore, the biological variability between samples will lead to experimental noise and the need for repetitions to obtain smooth kinetics. To make different phases in the re-oxidation kinetics visible, the use of a logarithmic time scale has been introduced (see e.g., Cser and Vass 2007). Commercial equipment to make this type of measurements is the superhead fluorometers (Photon Systems Instruments, Brno, Czech Republic), which can also be used to measure OJIP transients and saturating pulse protocols (see below).

Complementary techniques for flash fluorescence measurements are thermoluminescence (TL) (reviewed by Vass and Govindjee 1996; Misra et al. 2001a, b; Ducruet and Vass 2009) and delayed fluorescence (DF) (recently reviewed by Goltsev et al. 2009) measurements that provide specific information on recombination reactions within PSII RCs.

Flash fluorescence measurements are frequently used to study PSII mutants (e.g., Etienne et al. 1990; Nixon et al. 1991; Cser and Vass 2007) and can also be used in the case of treatments that affect the function of PSII [e.g., stresses like heat stress (Yamasaki et al. 2002)] or to probe the PQ redox state (Dannehl et al. 1996).

### Saturating pulse or OJIP measurements

Upon a dark-to-light transition, the fluorescence intensity of a leaf or other photosynthetic samples increases from a low value (*F*_O_ or O) via two intermediate steps (*F*_J_ or *J* and *F*_I_ or *I*) in 200–300 ms to a maximum value (*F*_M_ or P) during the application of a saturating pulse of light (see Fig. 3a, b; Strasser and Govindjee 1991; Strasser et al. 1995). The different fluorescence rise phases (OJ, JI and IP) can be related to different steps of the reduction of the ETC: OJ parallels the reduction of the acceptor side of PSII (*Q*_A_ + *Q*_B_); JI parallels the reduction of the PQ-pool and IP parallels the reduction of the electron transport acceptors in and around PSI (Schansker et al. 2005). This means that OJIP transients give information on the state of the ETC. Although complex simulations of OJIP transients use a kinetic model based on the gradual reduction of the ETC (see e.g., Lazár 2003; Zhu et al. 2005), it has been shown that the transients can also be approximated assuming that the transients consist of three kinetic components (Boisvert et al. 2006; Vredenberg 2008; Joly and Carpentier 2009) indicating that the rate limitations (exchange of PQ at the *Q*_B_-site of PSII and re-oxidation of PQH_2_ by cyt b_6_/f) quite effectively separate the three rise phases kinetically. The kinetics of the OJIP transient are, e.g., sensitive to the PQ redox state (Tóth et al. 2007a) and PSI content (Oukarroum et al. 2009; Ceppi et al. 2012). During the isolation of thylakoid membranes, the properties of the ETC are modified, and this is reflected by changes in the fluorescence kinetics. Attempts have been made (see e.g., Bukhov et al. 2003) to make the fluorescence induction kinetics of thylakoid membranes look more like those of leaves.

Using a pulse-probe approach, a first pulse reduces the ETC and a second probe pulse given at time *t* after the first pulse probes the redox state of the ETC. The analysis of the regeneration kinetics of the OJIP transient gives information on the rate of re-oxidation of *Q*_A_^−^ by recombination with the donor side of PSII, the re-oxidation of the PQ-pool due to plastoquinol oxidase activity (see Question 17), and the rate of re-oxidation of the acceptor side of PSI in darkness (Schansker et al. 2005).

Complementary techniques for OJIP measurements are 820 nm absorbance/transmission measurements that probe the redox state of PSI (plastocyanin, P700 and ferredoxin) and DF measurements that give information on the occurrence of recombination reactions in PSII as a function of the redox state of the ETC. The interpretation of these measurements can also be improved by determining the chl *a*/*b* ratio and the chl content of the leaves/cells. OJIP measurements have been used widely to study the effects of stress (see Questions 19, 24, 26–28).

### Steady state measurements

The steady state refers to the relatively stable photosynthetic activity that is obtained when leaves or other photosynthetic samples are illuminated at a chosen light intensity during approximately 5–10 min (or more). The Chl *a* fluorescence intensity in the steady state is affected both by the redox state of the ETC (and *Q*_A_ in particular) and by changes in the fluorescence yield, i.e., a change in the probability that absorbed light is emitted as Chl *a* fluorescence. These yield changes not only can be due to the formation of the transthylakoid ΔpH (Krause et al. 1983) and xanthophyll cycle (XC) related changes (Bilger and Björkman 1991), antenna size changes—for example, due to state transitions, which are especially obvious for algae such as *Chlamydomonas reinhardtii* (see e.g., Iwai et al. 2008)—or photoinhibition (see e.g., Björkman and Demmig 1987; Van Wijk and Krause 1991; Tyystjärvi and Aro 1996) but are also due to the activation of ferredoxin NADP^+-^reductase (FNR) on the acceptor side of PSI (Schansker et al. 2006, 2008). In the 1980s, an analysis was developed, called the quenching analysis (see Question 15 for a more detailed discussion of the quenching analysis) that can distinguish between redox changes (photochemical quenching) and fluorescence yield changes. A fluorescence yield change occurs when the rate constant for either fluorescence or heat emission changes. If this leads to a smaller *F*_M_ value (and in many cases smaller *F*_O_ value), this is called non-photochemical quenching. Figure 4 gives an example of such a protocol. Just as in the case of the flash fluorescence measurements (see above), the fluorescence intensity is probed using low-intensity modulated light. The steady state is induced using continuous actinic light of a chosen intensity, and in addition every 100 or 200 s (this can be variable time interval), a saturating pulse (comparable to an OJIP transient) is given to reduce the ETC and all *Q*_A_. On turning off the actinic light, relaxation of the induced non-photochemical quenching can be followed using saturating light pulses to probe changes in the *F*_M_ level. In general, three relaxation phases are observed (Demmig and Winter 1988; Horton and Hague 1988): the qE which relaxes within 100–200 s as a consequence of the dissipation of the transmembrane ΔpH, the qT, whose relaxation is complete within 15 min and the qI which covers all processes that need more than 15 min to recover. As will be discussed later in detail (see Question 15) the qT and qI are less well defined. It is worth mentioning here that by measuring Chl *a* fluorescence induced by the saturating pulses with a higher time resolution (i.e., measuring OJIPs), it is possible to obtain more information on the character of the qT and qI phases (Schansker et al. 2006). The relaxation of the different non-photochemical quenching phases can be treated as the sum of three exponentials (see e.g., Walters and Horton 1991; Roháček 2010; and Question 15).

Obtaining the ‘maximum’ *F*_M_′ value is not a trivial issue. Markgraf and Berry (1990) and Earl and Ennahli (2004) observed that in the steady state, high light intensities are needed to induce the maximum *F*_M_′ value. Earl and Ennahli (2004) observed that more than 7,500 µmol photons m^−2^ s^−1^ (the maximum intensity of their light source) were needed to reach the maximum *F*_M_′ value of their maize leaves and that at higher actinic light intensities, more light was needed to saturate *F*_M_′. Schansker et al. (2006) observed the same actinic light intensity dependence measuring both fluorescence and 820 nm transmission and suggested that the ferredoxin/thioredoxin system that is thought to continuously adjust the activity of several Calvin–Benson cycle enzymes (see Question 6), is responsible for the actinic light intensity dependence. Earl and Ennahli (2004) proposed an extrapolation method based on the measurement of *F*_M_′ at two light intensities to obtain the true *F*_M_′ value. Loriaux et al. (2013) studied the same light intensity dependence of *F*_M_′ and proposed the use of a single multiphase flash lasting approximately 1 s to determine the maximum *F*_M_′ value. This flash consists of two high light intensity phases separated by a short interval at a lower light intensity during which the fluorescence intensity decreases. The second high light intensity phase of this protocol has a higher light intensity than the first phase (see also Harbinson 2013 for a commentary on this paper).

Complementary techniques for this type of fluorescence measurement are gas exchange measurements (to probe Calvin–Benson cycle activity, stomatal opening, CO_2_ conductance) and 820 nm absorbance/transmission measurements.

### 77 K fluorescence spectra

**Fig. 5:**
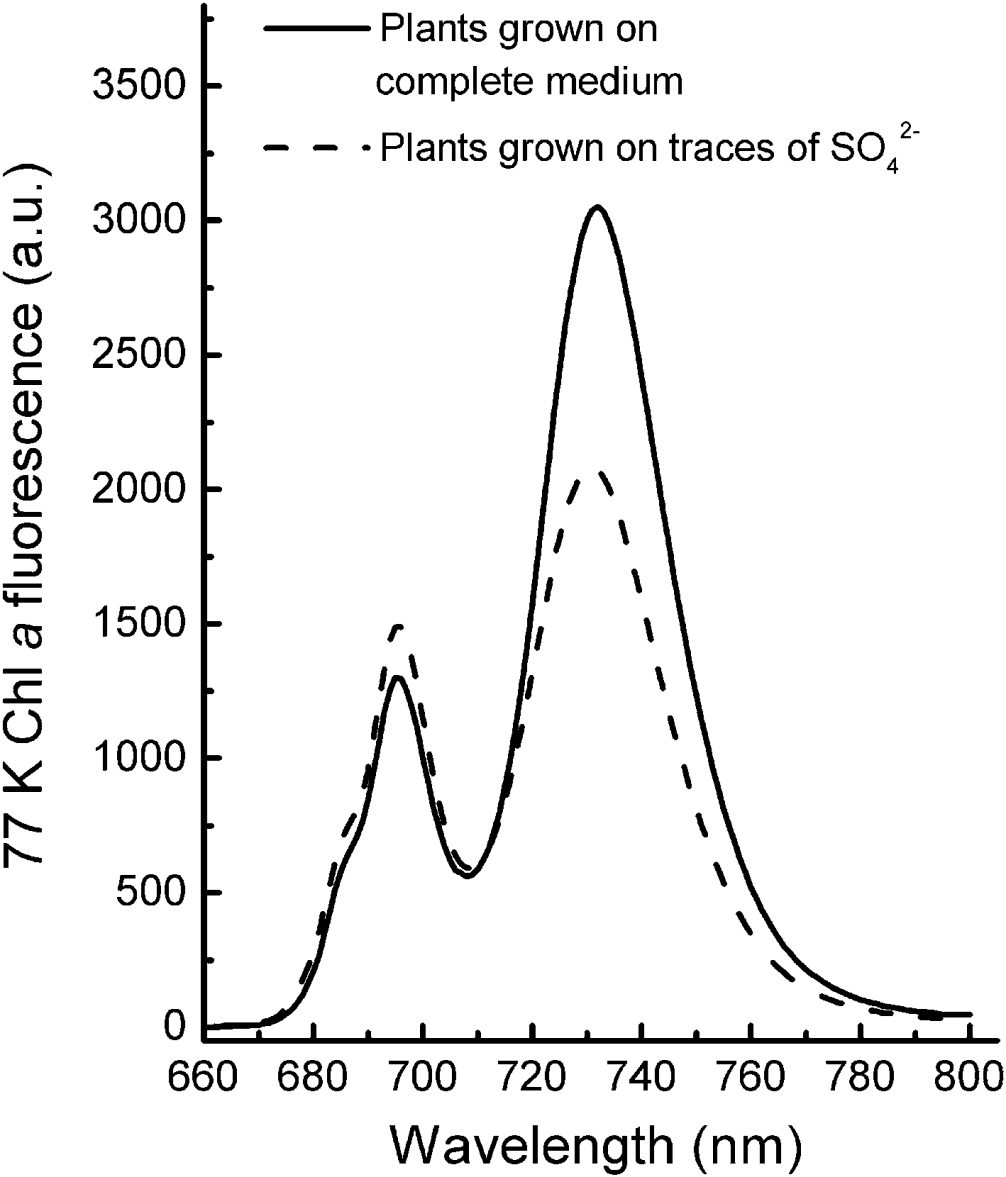
77 K fluorescence emission spectra of leaves of plants grown hydroponically on a complete medium (*black line*) and on medium containing only traces of sulfate (*green line*). Sulfate deficiency led to extensive chlorosis and in addition to a rather specific loss of PSI. This reduced the long wavelength bands around 730 nm and increased the 685 and 695 bands due to a decreased re-absorption by PSI reaction centers of Chl *a* fluorescence emitted by PSII (Schansker and Ceppi, unpublished data)

Low temperature (77 K) fluorescence measurements represent another technique to obtain information on the photosystems. At room temperature, variable fluorescence is emitted nearly exclusively by PSII. Byrdin et al. (2000) detected only a small difference in the quenching efficiencies of P700 and P700^+^ at room temperature. This is supported by the observation that inhibiting PSII by DCMU (Tóth et al. 2005a) or cyt b_6_/f by DBMIB (Schansker et al. 2005) does not affect *F*_M_ despite a big difference in the redox state of P700 in the absence and presence of inhibitors. However, variable fluorescence emitted by PSI can be induced on lowering the temperature to 77 K. Although measurements of light-induced fluorescence changes can be made at 77 K, in most cases, the fluorescence emission spectrum (600–800 nm) is measured. This type of measurement is used to obtain information on the PSII and PSI antennae. A common application of 77 K measurements is the detection of the occurrence of state transitions (e.g., Bellafiore et al. 2005; Papageorgiou and Govindjee 2011; Drop et al. 2014), where changes in the relative amplitudes of the PSII and PSI bands are indicators for this process. Figure 5 gives an example of a measured 77 K spectrum. Emission bands at 685 and 695 nm are related to the antenna of PSII, and peaks around 730 nm are related to the antenna of PSI (Govindjee 1995; Špunda et al. 1997; Srivastava et al. 1999).

Complementary techniques are ultrafast femto- or picosecond absorbance or fluorescence measurements that give information on energy transfer within the antenna (e.g., Gilmore et al. 1998; Richter et al. 1999) but which are beyond the scope of this educational review.

### Fast fluorescence techniques (ns, ps, fs time range)

As noted in the previous paragraph, fast fluorescence (and absorption) techniques, which probe energy transfer between chlorophylls or between carotenoids and chlorophylls in the photosynthetic antennae and the charge separation processes in the RCs of PSII and PSI will not be discussed in this paper. See e.g., Holzwarth (1996, 2008) and Berera et al. (2009) for introductory reviews on the application of these methods.

## Question 3. What is the effect of wavelengths at which the fluorescence is measured on the character of the fluorescence signal?

Most commercial instruments measure Chl *a* fluorescence at wavelengths longer than 700 nm. At room temperature, at wavelengths longer than 700 nm, PSI becomes an important source of fluorescence emission. As shown by Genty et al. (1990) and Pfündel (1998) in C3 plants, about 30 % of the *F*_O_ emission is due to PSI fluorescence, and in C4 plants, this percentage is even higher (Pfündel 1998). This causes, e.g., a systematic underestimation of the *F*_V_′/*F*_M_′ value, which is used as a measure of the maximum quantum yield of PSII. Detecting Chl *a* fluorescence emission at wavelengths below 700 nm can considerably reduce this problem. However, in measuring equipment such as photosynthetic efficiency analyser (PEA) and HandyPEA instruments (Hansatech Instruments Ltd, UK) which use red LEDs with an emission peak around 650 nm, this would have led to an overlap between the actinic wavelengths and the detecting wavelengths. With the introduction of (strong) LEDs emitting at shorter wavelengths, e.g., in the blue (see e.g., Nedbal et al. 1999), it is now technically possible to avoid this overlap and to detect fluorescence below 700 nm. Interference of PSI fluorescence at wavelengths longer than 700 nm should be taken into account especially when measuring fluorescence parameters in the light-adapted state. Non-photochemical quenching induced in the light quenches the variable fluorescence (*F*_M_–*F*_O_) to a larger extent than *F*_O_ fluorescence. This makes the underestimation of the true *F*_V_′/*F*_M_′ value light intensity dependent as well, since a higher light intensity induces more non-photochemical quenching.

## Question 4. Which part of the leaf is probed and analyzed by a fluorescence measurement?

The leaf is optically complex. In a dorsiventral leaf, the palisade parenchyma cells have been shown to act as light guides, keeping the light more or less focused (Vogelmann and Martin 1993; Vogelmann et al. 1996). The lobed cells of the spongy mesophyll and the spaces that surround these cells, on the other hand, disperse the light (Vogelmann and Martin 1993). At the same time, there is a strong light gradient within the leaf (Vogelmann 1989, 1993). This means that the light intensity decreases rapidly as light penetrates into the leaf. As a consequence, illuminating and probing Chl *a* fluorescence emission on the adaxial surface of the leaf, chloroplasts located deep in the leaf will be excited by a much lower photon flux density than those located close to the adaxial side of the leaf (Terashima and Saeki 1985; Fukshansky and Martinez von Remisowsky 1992). At the same time, the spectral distribution of the light changes as well: as light penetrates the mesophyll, the relative contribution of green and far-red (FR) light progressively increases, because the absorption of these wavelengths by the leaf is less efficient (Sun et al. 1998; Rappaport et al. 2007). The chloroplasts located deeper in the leaf, i.e., those of the spongy tissue, acclimate to these lower, FR-enriched light intensities by increasing the antenna size of PSII, reducing the number of RCs, and decreasing the PSI/PSII ratio (Terashima et al. 1986; Evans 1999; Fey et al. 2005; Pantaleoni et al. 2009). Since the emitted fluorescence is a linear function of the light intensity (Vogelmann and Evans 2002; cf. Schansker et al. 2006), chloroplasts located deeper in the leaf will contribute to a lesser extent to the detected fluorescence signal. In practice, fluorescence measurements will probe mainly chloroplasts in the palisade parenchyma cells (Vogelmann and Evans 2002). The assumption that not all chloroplasts are assayed is supported by the observation that a fivefold decrease in the chlorophyll content of the leaf does not affect the detected *F*_O_ and *F*_M_ values (Dinç et al. 2012). In fact, since the total amount of fluorescence emitted by the leaf does not change, it suggests that the light beam probes deeper in the leaf as more chlorophyll is lost. The optical properties of the leaf also mean that measurements made on the abaxial (bottom) side of the leaf have characteristics that differ considerably from those made on the adaxial (top) side of the leaf (Schreiber et al. 1977). Oxygen and CO_2_ assimilation measurements on the other hand assay the whole leaf, and this may lead to deviations when comparing, for example, measurements of the oxygen evolving activity with fluorescence measurements (Björkman and Demmig 1987; Tyystjärvi and Aro 1996).

Given the gradient of photosynthetic properties that exists within the leaf (Terashima et al. 1986; Evans 1999), the photosynthetic response of a leaf depends on the wavelength composition of the exciting light. Deeper penetrating green light probes more low light acclimated chloroplasts located in the lower cell layers than blue light that is strongly absorbed by the leaf and mainly probes chloroplasts close to the adaxial side of the leaf.

## Question 5. How to dark-adapt leaves?

For the interpretation of Chl *a* fluorescence measurements, it is important that the state of the photosynthetic apparatus at the beginning of the measurement is well defined. The dark-adapted state of the leaf is a well-defined state of the photosynthetic apparatus and, therefore, for most experiments, photosynthetic samples are first dark adapted.

There are four main methods to achieve dark adaptation in leaves:

1. In the case of an intact plant, a leaf can be put into a leaf clip shielding it from ambient light. However, if the ambient light intensity is high, and the leaf is not entirely flat, there is a chance that some stray light reaches the shielded area.
2. Detached leaves can be kept for a while between wet filter paper in darkness and subsequently measured in the laboratory. Detachment of leaves has consequences for the physiological state of the leaf: it causes, for example, a closure of the stomata (Raschke 1970). See Potvin (1985) and Weng et al. (2011) for a comparison of the properties of attached and detached leaves and Kato et al. (2002) for a discussion of the differences between leaves and leaf disks.
3. Under laboratory conditions, measurements can be made in the dark or in a dimly lit room under conditions that induce very little photosynthetic activity. Traditionally, low-intensity green light has been used as a kind of safe light (see Sun et al. 1998 for a discussion of this point) although we note that leaves can still absorb and use most of the green light for photosynthesis (cf. Sun et al. 1998; Vogelmann and Evans 2002; Rappaport et al. 2007).
4. Loss of time for dark adaptation can be avoided when the measurements are made directly in the field at night (no need for leaf clips). In this case, the leaves are allowed to dark adapt for many hours, and the results of such measurements differ from measurements on leaves following a relatively short dark-adaptation period during the day.

## Question 6. What is a “good” dark-adaptation time?

Dark adaptation of samples that will be used for Chl *a* fluorescence measurements, is often associated with the re-oxidation of *Q*_A_^−^. However, dark adaptation is a considerably more complicated process, and there are more factors that can affect a subsequent fluorescence measurement.

In dark-adapted leaves, several enzymes are inactivated to prevent wasteful reactions. Examples of such enzymes include Rubisco (e.g., Streusand and Portis 1987); four other thioredoxin-dependent enzymes: d-fructose1,6-bisphosphatase, phosphoribulokinase, and sedoheptulose-1,7-bisphosphatase (Buchanan 1984; Scheibe 1990) and ATP synthase (Stumpp et al. 1999); and FNR (Carillo et al. 1981; Satoh 1981). These enzymes are active in the light, and during a light-to-dark transition, they gradually become inactive again. The half-time of inactivation of Rubisco under in vivo conditions is 2–4 min (Stitt et al. 1987; Laisk and Oja 1998). Inactivation of ATP synthase and the three other Calvin–Benson cycle enzymes is under control of the thioredoxin system (Scheibe 1990), and their inactivation depends on the re-oxidation of stromal components such as ferredoxin and NADPH. FNR inactivation varies depending on the species: pea leaves need ~15 min for full inactivation (Schansker et al. 2006), whereas in a *Pinus* species, an hour is needed (Schansker et al. 2008). Once inactivated, all of these enzymes must first be activated again before steady state photosynthesis is induced, and this affects the fluorescence induction kinetics (see Papageorgiou et al. 2007; Papageorgiou and Govindjee 2011 for an in-depth discussion of the fluorescence kinetics beyond P or *F*_M_ in a variety of photosynthetic organisms). In addition, active FNR (i.e., an activated acceptor side of PSI) has an effect on the IP phase of the OJIP transients and on the amplitude of the *F*_M_ that can be reached by a strong pulse of light (Schansker et al. 2008). In most fluorescence studies, many are not interested in the processes mentioned above, and in that case, it is best to make the dark-adaptation time long enough to allow at least FNR to become inactive again (a marker for this is a regeneration of the fluorescence IP phase and in addition a regeneration of 820 nm re-reduction phase paralleling the IP phase, see Schansker et al. 2006, 2008).

As mentioned in Question 2 Sect. 3, several regulatory and stress-related processes that affect the fluorescence yield (quench *F*_M_) are induced in the light. Following a light-to-dark transition, i.e., on turning off the light, these processes are reversed. State transitions (the transfer of a part of the antenna system among PSII and PSI) and XC related processes may take a considerable amount of time to reverse (Fork and Satoh 1986; Ruban and Horton 1999) and the recovery of a plant from photoinhibition takes hours (Havaux 1989; Long et al. 1994).

An answer to the question as to what a good dark-adaptation time is, depends on the information we want to obtain. If the aim is the study of the regulatory and photoinhibition-related processes, a dark-adaptation time of 15 min that allows FNR (at least in plants like pea) to become inactive again would be sufficient. If someone is interested in long term adaptation responses of a leaf or other photosynthetic organism to a treatment, much longer dark-adaption times that allow also the regulatory processes and processes like photoinhibition to recover may be considered (see also the next question).

## Question 7. How to obtain the best reference *F*_O_ and *F*_M_ values for the quenching analysis?

In field experiments, predawn measurements are often used to obtain reference *F*_O_ and *F*_M_ values for measurements made during the day (Logan et al. 1999; Maxwell and Johnson 2000; Demmig-Adams et al. 2006). Under these conditions, NPQ is assumed to be completely relaxed and therefore zero, and the photoinhibition induced during the previous day is expected to have been reversed (Flexas et al. 1998; Logan et al. 1999; Demmig-Adams et al. 2006). However, in some cases, chronic photoinhibition occurs, which can be easily detected by lowered predawn *F*_V_/*F*_M_ values (Osmond and Grace 1995; for a review see Demmig-Adams et al. 2012). We note that the absence of light during recovery experiments may prevent a full repair of photoinhibitory (Greer et al. 1986) and heat stress damage (Tóth et al. 2005b). Light is needed for the synthesis of ATP, which is needed for the synthesis of the D1 protein (Kuroda et al. 1992). Edhofer et al. (1998) have reported that light is needed for translation elongation of the D1 protein; these are processes that are part of the PSII repair cycle following damage to PSII (recently reviewed by Nixon et al. 2010). Low-intensity actinic light generates the ATP needed for the PSII repair cycle, and at the same time, it does not induce additional photoinhibition and is thereby more effective than a complete dark recovery (see e.g., Elsheery et al. 2007).

## Question 8. What can go wrong during a fluorescence measurement on leaves? Technical issues

To dark-adapt leaves in the field, leaf clips have been developed. They cover the area of the leaf to be measured. The measuring head of, for example, a HandyPEA can be connected to a leaf clip, after which the clip can be opened, and the measurement made. Since such measurements are normally evaluated afterward, it should be kept in mind that unopened or partially opened leaf clips are a common reason for transients showing no or little fluorescence rise. A smooth leaf can also lead to problems, since the clip may shift while attaching the measuring head, and in that case, a non-dark-adapted part of the leaf will be measured. If the leaf is not flat, some stray light may enter the leaf clip via the spaces left between the leaf clip and the leaf surface. Especially on a bright day, this may prevent a full dark adaptation of the covered leaf area. The same problems can occur with pulse amplitude modulated (PAM) type instruments developed for field applications, which use leaf clips to allow dark adaptation.

When working with a PAM instrument, the measuring light intensity must be chosen in such a way that the *F*_M_ stays within the measuring window. If the measured signal is too strong, then the highest values will be cut off. For example, as a rule of thumb the fluorescence intensity induced by the measuring light (associated with *F*_O_) should be approximately 10 % of the total scale. In any case, absolute values and their limits depend on the manufacturer, and its instructions should be carefully read before starting any measurements. Further, the distance between the leaf and the fiber optics has to be adjusted; it is usually set between 1 and 1.5 cm. Background fluorescence signals from the environment must be suppressed by zeroing the signal in the absence of a leaf sample.

Using direct fluorescence equipment like the HandyPEA, there is also a risk that the emitted fluorescence intensity causes an overload of the detector. It is therefore important to check if, at a given gain and excitation light intensity, the measured fluorescence kinetics remain below the maximum measurable fluorescence intensity. If the emitted fluorescence intensity is too strong, then the top part of the transient will be cut off, and in that case, the gain has to be reduced.

## Question 9. Why was it so difficult to determine the *F*_O_ before ~1985?

It may be hard to imagine nowadays, but the determination of a correct F_O_ value was a major problem for researchers using Chl *a* fluorescence up to the mid-1980s (see Kalaji et al. 2012a, b for a historical overview of instrument development). The shutters used at the time had a full opening time of anywhere between 0.8 ms (e.g., Neubauer and Schreiber 1987) and 2 ms. At high light intensities, the J-step is reached after ~0.8–2 ms of illumination. To minimize the effect of the shutter opening time, in many studies, low-intensity light was used to slow down the fluorescence induction kinetics. In the 1980s, two fundamentally different solutions for the shutter problem were introduced in the form of modulated systems (Schreiber et al. 1986) and PEA-type instruments (Strasser and Govindjee 1991). These two measuring concepts are explained and compared in Questions 10 and 11.

## Question 10. What is the principle of modulated fluorescence measurements?

Modulated systems, pulse amplitude modulated fluorometers, (PAM) use a trick to separate the effect of the actinic light that drives photosynthesis and the low-intensity measuring light that is used to probe the state of the photosynthetic system on the measured fluorescence intensity (see also Question 2 Sect. 3). A so-called lock in amplifier only registers the fluorescence changes induced by the modulated measuring light and ignores the fluorescence changes induced by the continuous actinic light. This way the low-intensity measuring light can be used to measure both the *F*_O_ (induced by the measuring light itself) and *F*_M_ (induced by a strong light pulse) values (Schreiber et al. 1986). The effective light intensity of modulated light depends on the pulse frequency. In the case of a modern PAM instrument, the modulated measuring light consists of 1–3 µs flashes of red or white light, and flash frequencies between 100 and 20,000 Hz can be chosen. At the lowest frequency, the effective photosynthetic photon flux density is <0.2 µmol photons m^−2^ s^−1^; an intensity that is 200 times higher when the highest frequency is chosen. The choice of a low frequency gives not only a very small actinic effect (= measuring-light-induced *F*_V_) but also a relatively poor signal-to-noise ratio. A high frequency not only is considerably more actinic but gives also a much better signal-to-noise ratio. The actinic effect of the measuring light becomes especially visible (and problematic) if PSII electron transfer inhibitors such as DCMU are being used (see Question 2 Sect. 1). Compared to PEA-type instruments an advantage of the modulated fluorimeters is that the measured fluorescence yield is independent of the intensity of both the actinic light and light of the saturating pulse (Schreiber et al. 1986). In the case of PEA-type instruments, the measured fluorescence intensity is a linear function of the actinic light intensity used, and as a consequence, the measured fluorescence intensities must be normalized first (e.g., divided by the light intensity) before measurements made at different light intensities can be compared (see e.g., Schansker et al. 2006).

## Question 11. What is the principle of direct fluorescence measurements?

In the so-called direct fluorescence instruments-i.e., instruments in which the actinic light that drives photosynthesis is also used as measuring light-the *F*_O_ problem is solved by using strong light emitting diodes (LEDs): light sources that can be switched on/off very quickly (Strasser and Govindjee 1991). In modern equipment, a stable light intensity emitted by the LEDs is reached in less than 10 μs. Initially, only red (650 nm) LEDs were available for this type of measurement but now colors like other orange (discussed by Oxborough 2004), green (Rappaport et al. 2007), and blue (Nedbal et al. 1999) or a mix of LEDs of different colors (Schreiber 1998) are also available. In the original PEA instrument, the response time of the LEDs was still in the order of the 40–50 μs (e.g., Strasser et al. 1995) necessitating the use of extrapolation to estimate the *F*_O_ value; in the current instruments, a response time of 10–20 μs is good enough for an accurate determination of the *F*_O_ value for light intensities below ~10,000 μmol photons m^−2^ s^−1^ (cf. Schansker et al. 2006). The absence of a measuring light source means that between pulses, there is true darkness. As a consequence, the *F*_O_ can be determined more accurately than in the case of a modulated system (see Schansker and Strasser 2005 for a discussion on the effects of very low light intensities on the *F*_O_ value). The absence of measuring light is particularly advantageous when the samples to be analyzed have been inhibited with electron transfer inhibitor such as DCMU. Another important difference between PEA instruments and modulated PAM instruments is the data sampling strategy. In PEA instruments, the data sampling is non-linear. In HandyPEA instruments, during the first 300 μs of illumination one measuring point is collected every 10 μs; between 300 μs and 3 ms one point per 100 μs, between 3 and 30 ms one point per ms, and between 30 and 300 ms one point per 10 ms. In this way, an OJIP transient measured at a high time resolution is defined by approximately 120 measuring points. In the case of a PAM instrument, a measurement with the same initial time resolution would yield at least 20,000 measuring points (for 200 ms). This makes the HandyPEA files much easier to handle when analyzing them using spreadsheet programs like Microsoft Excel.

## Question 12. Why use a logarithmic timescale to visualize fluorescence transient measurements?

As described above, PEA instruments allow a shutter-less measurement of OJIP transients. However, PEA instruments make use of a second innovation and that is the use of a logarithmic timescale to visualize the measurements of the OJIP fluorescence rise (Strasser and Govindjee 1991). Bannister and Rice (1968) had already used this idea more than 20 years earlier, but at that time, it was not picked up by others. The logarithmic timescale was later exploited by researchers measuring fluorescence relaxation following a STF, as well (see Question 2 Sect. 1; e.g., Cser and Vass 2007). The logarithmic time scale distorts the time dependence somewhat but, at the same time, allows the visualization of considerably more kinetic features than is possible on a linear time scale. This additional kinetic detail makes it much easier to detect changes in the fluorescence kinetics. Fluorescence measurements shown on a linear timescale are always dominated by the slower changes (see Fig. 3a). A logarithmic timescale turns exponential rise phases into sigmoidal rise phases, and we must keep in mind that the sigmoidicity of the fluorescence rise cannot be derived on the basis of fluorescence transients visualized on a logarithmic timescale.

## Question 13. Direct or modulated fluorescence?

It is possible to measure OJIP transients using a modulated system (Schreiber 1986; Neubauer and Schreiber 1987; Schreiber and Neubauer 1987), and at the same time, it is possible to make a quenching analysis (see Questions 2.3 and 15) using a PEA-type instrument (Schansker et al. 2006). However, modulated instruments are much better suited for a quenching analysis, and PEA-type instruments are the instruments of choice for a study of the OJIP kinetics. Thus, we recommend that both must be used to get a complete picture.

## Question 14. What kind of additional information can be obtained using fluorescence imaging?

All the instruments, discussed thus far, integrate the signal of the measured area. Fluorescence imaging permits the study of spatial heterogeneities in the fluorescence emission intensity within cells, leaves, or whole plants; heterogeneities caused by a range of internal plant factors (Gorbe and Calatayud 2012). It can also be used to average and analyze the fluorescence signal from much larger leaf areas than classical methods would allow, and at the same time, it allows the simultaneous measurement/screening of many samples/mutants in, for example, a microwell plate or of colonies grown on a Petri dish (see e.g., Niyogi et al. 1997; Serôdio et al. 2012) or all the leaves of an rosette of *Arabidopsis*. There are several commercial imaging instruments on the market. It is a technique whose development has kept pace with improvements in LED technology. For reliable imaging measurements, it is critical that the whole sample area be illuminated homogeneously. Several introductory texts and reviews have been published on fluorescence imaging (e.g., Buschmann et al. 2001; Oxborough 2004; Lenk et al. 2007; Scholes and Rolfe 2009). Since it was not possible to image *F*_O_′ with the imaging systems available in the late 1990s, Oxborough and Baker (1997) derived an equation to estimate it:

$$ F_{\text{O}}{'} =\, \frac{{F_{\text{O}} }}{{\frac{{F_{\text{V}} }}{{F_{\text{M}} }} + \frac{{F_{\text{O}} }}{{F_{\text{M}} {'}}}}}. $$

This equation allows the calculation of the parameters qP [=(*F*_M_′ − *F*_S_)/(*F*_M_′ − *F*_O_′)] and *F*_V_′/*F*_M_′. The challenge using fluorescence imaging is to process all the data collected in a scientifically meaningful way. Meyer and Genty (1998) analyzed their data making frequency distributions of parameters of interest; we recommend that this method is considered for future experiments.

Imaging can be used, e.g., to assess the dynamics and heterogeneous behavior of stomatal opening/closure over a leaf, a phenomenon also called stomatal patchiness. A palette of false colors is used to cover the range of fluorescence intensities (normalized between 0 and 1), assigning a color to each pixel of the image (Gorbe and Calatayud 2012). Based on the image, different areas of the leaf can be chosen, the associated fluorescence data averaged, fluorescence parameters can be calculated, and subsequently, the photosynthetic properties of the chosen area can be studied.

Using fluorescence imaging, it is easy to detect photosynthetic heterogeneities in a leaf (Meyer and Genty 1998) and to follow how any stress affects the leaf in spatial terms. In a popular early experiment, the imaging technique was used to show the gradual infiltration of PSII inhibiting herbicides in the leaf (e.g., Daley et al. 1989; Lichtenthaler et al. 1997; Chaerle et al. 2003) or the effect of reactive oxygen species (ROS)-inducing herbicides (e.g., Hideg and Schreiber 2007). Spatial heterogeneities that have been studied using fluorescence imaging include heterogeneities occurring during the following processes: induction of photosynthesis (Genty and Meyer 1995; Daley et al. 1989), the onset of senescence (Wingler et al. 2004), chilling (Hogewoning and Harbinson 2007), the response to drought (Woo et al. 2008), nutrient stress (Landi et al. 2013), ozone stress (Gielen et al. 2006; Guidi et al. 2007), wounding (Quilliam et al. 2006), and during infection with viruses (Balachandran et al. 1994) or fungi (Guidi et al. 2007). Several studies, using imaging to study Chl *a* fluorescence parameters under various conditions (high/low ambient CO_2_ concentration, high/low light intensity, etc.), have yielded information on the relationship between leaf structure and organization on the one hand and the response to stress conditions on the other (Baker 2008; Roháček et al. 2008; Guidi and Degl’Innocenti 2011; Gorbe and Calatayud 2012).

Serôdio et al. (2013) have introduced, a new application of fluorescence-imaging systems, which allows the rapid generation of light-response curves (see Question 18) simultaneously illuminating replicates of samples using spatially separated beams of actinic light of different intensities.

## Question 15. What kind of information can be obtained using the quenching analysis (see Question 2)?

In leaves exposed to a certain irradiance, the fluorescence intensity is affected by changes both in the redox state of the ETC (particularly the redox state of *Q*_A_) and in the fluorescence yield due to light-induced changes in the properties of the PSII antenna. A method called the quenching analysis was developed to separate these two types of process. In most cases, the quenching analysis is used to describe the steady state, i.e., the stable photosynthetic activity, which is usually reached after approximately 5–10 min of illumination at a chosen actinic light intensity.

A protocol was developed (Schreiber et al. 1986; Fig. 4) based among others on the work of Bradbury and Baker (1981) in which the measurements are initiated by switching on the measuring light to determine the *F*_O_ value of a dark-adapted sample. A saturating light pulse is then applied to determine the *F*_M_. The measurement is continued switching on an actinic light source to induce photosynthesis, until the fluorescence emission stabilizes at a level called *F*_S_. The *F*_M_′ is then determined by applying another strong pulse of light followed some time later (e.g., 10 s) by turning off the actinic light. Turning off, the actinic light will cause a quick, partial, re-oxidation of the photosynthetic ETC. Within the first 100 ms of darkness, the PQ-pool will be largely re-oxidized by forward electron transport toward PC^+^ and P700^+^, and a value close to *F*_O_′ can be measured. The *F*_O_′ level subsequently increases again due to non-photochemical reduction of the PQ-pool by NADPH and possibly *F*d_red_ (Mano et al. 1995; Gotoh et al. 2010; Guidi and Degl’Innocenti 2012). This so-called “*F*_O_′ rise” can be almost completely suppressed by a short pulse of FR light (e.g., of 1 s duration) following the turning off of the actinic light. The increase of the fluorescence intensity from *F*_S_ to *F*_M_′ is related to a change in the redox state of the ETC, whereas the difference between *F*_M_′ and the dark-adapted *F*_M_ is then a measure of the fluorescence yield change, which in the case of qE is associated with increased heat dissipation. In quenching analysis terminology, this approach splits the fluorescence changes into a photochemical quenching (redox related) and a non-photochemical quenching (fluorescence yield related) part. On turning off the actinic light, the relaxation of the non-photochemical quenching, i.e., the increase of *F*_M_′ to *F*_M_, can be followed and several contributing processes can be resolved (Walters and Horton 1991; Roháček 2010). Schreiber et al. (1986) introduced the parameter qN = 1 − *F*_V_′/*F*_V_ to quantify changes in the non-photochemical quenching. The parameter qN can range between 0 and 1, and for its calculation, the *F*_O_′ value is needed. In 1990, Bilger and Björkman (1990) introduced the parameter NPQ = *F*_M_/*F*_M_′ − 1 which has as advantages over the parameter qN that its range is not restricted (see Question 21), and in addition, it is not necessary to know the *F*_O_′ value. However, Holzwarth et al. (2013) evaluating the parameter NPQ, concluded that in this treatment of the fluorescence data, the relationship between the quenching parameter and the underlying processes becomes distorted, especially when the time dependence of NPQ is considered.

For the analysis of the relaxation kinetics of the parameter qN semi-logarithmic plots of Log(q*N*) versus time are made. This linearizes the slowest component. Using linear regression, the decay half-time and amplitude of this component can be determined. This component (an exponential function) can then be subtracted from the original data, and a new semi-logarithmic plot can be made of the remaining q*N*. The procedure can then be repeated (e.g., Walters and Horton 1991; for a discussion of the theoretical basis of the resolution method, see Roháček 2010).

The least controversial of these kinetic processes is the process relaxing during the first 100–200 s of darkness, with a relaxation half-time of ~30 s. In quenching analysis terms, this is called the qE or high-energy quenching; it depends on a low lumen pH and is affected by the XC (reviewed by Horton et al. 1996; Müller et al. 2001; Gilmore 2004; Krause and Jahns 2004; Ballottari et al. 2012). However, the exact mechanism of the induction of the qE and the exact components involved in this process are still a hotly debated issue (e.g., Caffari et al. 2011; Johnson et al. 2011; Miloslavina et al. 2011). A set of mutants has been generated playing an important role in the study of the q*E*, in which different components and processes related to qE have been modified (Niyogi et al. 1998). The second process, the qT, with a half-time of 5–10 min has been assigned to state II to state I transitions (transfer of LHCII units from PSI to PSII) based on the observation that it was already induced at low light intensities (Demmig and Winter 1988) and on its possible sensitivity to the phosphatase inhibitor NaF (Horton and Hague 1988). Schansker et al. (2006) studying the kinetics of the saturating pulses showed that the main fluorescence change occurring in this time interval in pea leaves is the regeneration of the IP phase suggesting that the qT reflects the inactivation of the acceptor side of PSI (the inactivation of FNR). Other processes that have been associated with the qT are some slowly relaxing component(s) of qE (Lokstein et al. 1993; Joliot and Finazzi 2010) and light-dependent movements of chloroplasts (Cazzaniga et al. 2013). In practice, there are several arguments making it doubtful that the qT is a reliable measure for state transitions. The slowest relaxation phase, the qI, which may last several hours can consist of several processes: photoinhibition of PSII and XC related changes (reviewed by Krause and Jahns 2004) and possibly also state II to state I transitions (Schansker et al. 2006) if a change in the JI amplitude is related to state transitions as suggested by Schreiber et al. (1995) for cyanobacteria. It should be noted that the rate with which these processes reverse in darkness is not necessarily the same in all photosynthetic organisms. For example, the regeneration of the IP phase parallels the qT phase in pea leaves (Schansker et al. 2006), and it is complete within 15 min, whereas the same process in needles of *Pinus halepensis* takes 1 h (Schansker et al. 2008).

## Question 16. Why is far-red light used to determine the *F*_O_ and *F*_O_′ values?

For leaves, it is reasonable to assume that under most conditions, nearly all PSII RCs are in the open state (*Q*_A_ oxidized) following dark adaptation. However, the assumption is not true for heat-stressed leaves (Ducruet 1999; Tóth et al. 2007b) and leaves that show a high rate of chlororespiration. Chlororespiration refers to the non-photochemical reduction of the plastoquinone pool by reducing equivalents derived from Fd_red_ or NADPH in the stroma (Bennoun 2002). Feild et al. (1998) showed a high chlororespiratory activity in light acclimated sunflower leaves following a light-to-dark transition leading to considerably higher *F*_O_′ values. This *F*_O_′ increase is due to a population of reduced *Q*_A_ associated with a more reduced PQ pool. There is redox interaction between the PQ-pool and *Q*_A_ leading to a redox-equilibrium (Diner 1977); for pea leaves, it was shown that a completely reduced PQ-pool (induced by anaerobiosis) is in equilibrium with reduced *Q*_A_ in 20 % of the PSII RCs (Tóth et al. 2007a).

To assure maximum oxidation of the PQ pool, the leaf can be pre-illuminated with FR light. For this purpose, FR light in the 720–735 nm range is normally used. FR light preferentially excites PSI and thereby causes an oxidation of the PQ pool. We note that FR light can induce charge separations in PSII (Pettai et al. 2005; Schansker and Strasser 2005). Pettai et al. (2005) demonstrated that FR light at 740 nm still induces a low level of oxygen evolution even though the activity is three times less than that induced by FR light at 720 nm. In practice, FR light induces about 2.5 % of *F*_V_ associated with *Q*_B_^−^ in 50 % of the RCs (Schansker and Strasser 2005). However, this observation is only of importance for direct fluorescence measurements, since the effects induced by FR light are also induced by the measuring beam of a modulated fluorescence instrument.

A short FR pulse (~1 s, at ~720–735 nm) given to a light-adapted leaf has two main effects: (i) it re-oxidizes the PQ-pool within 100 ms and (ii) it suppresses the transient *F*_O_′ increase, which is normally observed following a light-to-dark transition (Mano et al. 1995; Gotoh et al. 2010; Guidi and Degl’Innocenti 2012). It is related to non-photochemical reduction of the PQ-pool by NADPH or Fd_red_; this process is mediated by an enzyme complex called NADPH dehydrogenase (NDH) (Burrows et al. 1998). The induction of the qE component of non-photochemical quenching leads to a quenching of the *F*_M_ level and in many plant species to a quenching of the *F*_O_′ level as well (Bilger and Schreiber 1986; Bilger and Björkman 1991; Noctor et al. 1991). This qE quenching relaxes quickly in darkness. To determine the associated *F*_O_′ quenching accurately, the *F*_O_′ level must be determined immediately after turning off the actinic light. The non-photochemical reduction of the PQ-pool affects the *F*_O_′ level as well, and this may complicate an accurate determination of the extent of *F*_O_′ quenching. Since the non-photochemical reduction of the PQ-pool is a rather slow process peaking approx. 40 s after turning off the light (Burrows et al. 1998), and the maximum re-oxidation of the PQ-pool following lights off takes less than 100 ms (Ceppi 2010), the *F*_O_′ level can be determined quite accurately before the transient non-photochemical reduction of the PQ-pool sets in. However, using ~1 s of FR is the most straightforward approach to obtain an oxidized PQ pool.

## Question 17. How can the NPQ index be calculated when NPQ is formed in the dark?

As noted in Question 16, a process called chlororespiration has been identified in higher plants (Bennoun 1982, 2002; Rumeau et al. 2007). Cyanobacteria, which are thought to be the ancestors of the chloroplast, lack mitochondria; instead they have a respiratory chain that shares the PQ-pool with the photosynthetic ETC (Vermaas 2001; Schmetterer and Pils 2004; Hart et al. 2005). It allows the creation of a pH gradient over the thylakoid membrane in the dark, and this gradient is utilized to synthesize ATP. In the dark, the respiratory activity in cyanobacteria is considerably higher than in higher plants. In fact, chlororespiration in higher plants is seen as a rudiment of the original respiratory chain. Also in green algae, the respiratory chain is still quite active (see Beardall et al. 2003 for a discussion of this topic). Another group of organisms that have been shown to have a high chlororespiratory activity are some microalgae, including diatoms (e.g., Caron et al. 1987). As a consequence, there is no complete relaxation of qE in the dark. XC activity in dark grown diatoms occurs as a result of the acidification of the thylakoid lumen due to this chlororespiratory activity (Jakob et al. 1999).

One effect of this high chlororespiratory activity in diatoms is that the *F*_M_ level of dark-adapted diatoms is lower than the *F*_M_′ observed under low actinic light (Cruz et al. 2010). This means that it is not possible to apply the commonly used NPQ equation:

1 $$ {\text{NPQ}} = \frac{{F_{\text{M}} }}{{F_{\text{M}} {'}}} - 1, $$

since the calculated value would be negative [*F*_M_ < *F*_M_′]. A practical solution for this problem is the determination of the light-response curve (see Question 18) and to replace *F*_M_ by the maximum *F*_M_′ level measured (*F*_M_′_max_; Serôdio et al. 2006) in Eq (1):

So,

2 $$ {\text{NPQ}}\; = \;\frac{{F_{{{\text{M }}\hbox{max} }} ^{\prime}}}{{F_{\text{M}} {'}}} - 1. $$

In this way, NPQ values will always be positive and approach a minimum value close to zero under conditions closely corresponding to a state with a very small transthylakoid proton gradient.

## Question 18. Can the time that is needed for a complete quenching analysis be shortened?

To characterize the properties of parameters such as qP, *Φ*_PSII_ [= (*F*_M_′ − *F*_S_′)/*F*_M_′] and NPQ, it is common practice to determine the light intensity dependence of these parameters (see e.g., Bilger and Björkman 1991; Gray et al. 1996; Verhoeven et al. 1997). The classical approach is to illuminate the leaf at each light intensity, until steady state is reached (see Questions 2.3 and 10). This process can be quite time-consuming, especially if the fluorescence quenching analysis is performed for field experiments.

To reduce the time needed for this type of measurement, a faster procedure was developed and called rapid light curves (RLCs) (White and Critchley 1999; Ralph and Gademann 2005). RLCs can be used to study the physiological flexibility of the photochemistry in response to rapid changes in irradiation (Guarini and Moritz 2009). Such changes occur frequently in natural environments. An RLC is a plot of the electron transport rate (ETR: *Φ*_PSII_ × PFD × 0.5 × leaf absorptivity coefficient) as a function of the actinic light intensity, which is applied for fixed short-time periods (e.g., 10 s or 1 min). Here, PFD stands for photon flux density, and here, it is assumed that the PSI:PSII ratio is 1:1. However, this is only a rough approximation and the real ratio will differ between samples (see Question 26). For this type of analysis, two criteria are important: (1) the samples must be dark adapted, and (2) photosynthesis must be induced [activation of the Calvin–Benson cycle enzymes that become inactive during incubation in darkness (see Question 6)] before the measurement sequence is started (White and Critchley 1999). Dark adaptation of the samples allows the determination of the reference *F*_O_ and *F*_M_ values needed for the calculation of qN and/or NPQ. If light-adapted samples are used for the experiments, for which reference *F*_O_ and *F*_M_ values are missing, then the effective quantum yield (*Φ*_PSII_) and ETR can still be calculated, but not the non-photochemical quenching parameters, nor qP. In other words, the best protocol consists of a dark acclimation of the sample, a weak modulated beam and a saturating pulse to determine the reference *F*_O_ and *F*_M_, respectively, and then a pre-illumination with a moderate light intensity (approx. 50 % of the ambient light intensity applied for several minutes is appropriate for this purpose) after which the RLC protocol is applied (see Lichtenthaler et al. 2005).

**Fig. 6:**
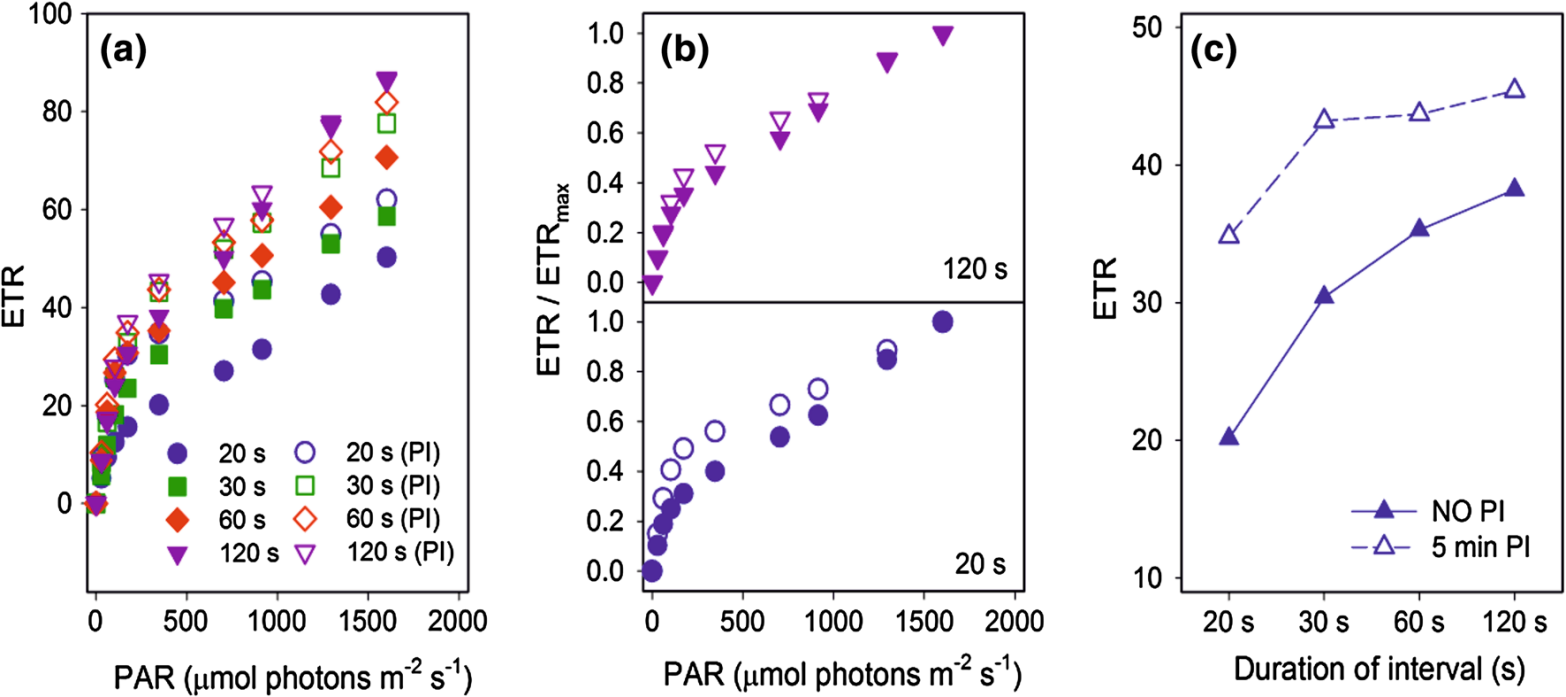
Rapid light curves. **a** Example of RLCs (PAR vs. ETR) for which the duration of light intervals (20, 30, 60, 120 s) had been varied. *Closed symbols* represent the values measured after 30 min dark acclimation (without pre-illumination), and open symbols represent values measured following 30 min of dark acclimation and 5 min of pre-illumination at a moderate light intensity (100 µmol photons m^−2^ s^−1^). **b** The ETR/ETRmax ratio (ETRmax represents the maximum value for each curve) of measurements with light intervals of 120 and 20 s. **c** ETR values of experiments without pre-illumination (NO PI) and with 5 min of pre-illumination (5 min PI, 350 µmol photons m^−2^ s^−1^). Measurements were made on *Citrus* leaves using a Dual-PAM fluorometer (Walz, Germany) (Brestič and Zivčak, unpublished data)

Examples of RLCs (Fig. 6a) illustrate the importance of the duration of light intervals. In addition to differences in the values determined for individual light intensities, there is also a difference in the shape of the curves (Fig. 6b). Pre-illumination at moderate light intensities ensures faster induction. Thus, in pre-illuminated samples, a 30-s interval is sufficient to obtain appropriate values and shapes of the curves that are comparable to those measured with 2-min intervals (Fig. 6c).

RLCs have frequently been used in studies dealing with plant stress (reviewed in Brestic and Zivcak 2013). The value of the RLC approach increases if a second technique, e.g., 820 nm or gas exchange measurements, is applied simultaneously, or if fluorescence-imaging measurements are also made.

## Question 19. What is the JIP test?

**Fig. 7:**
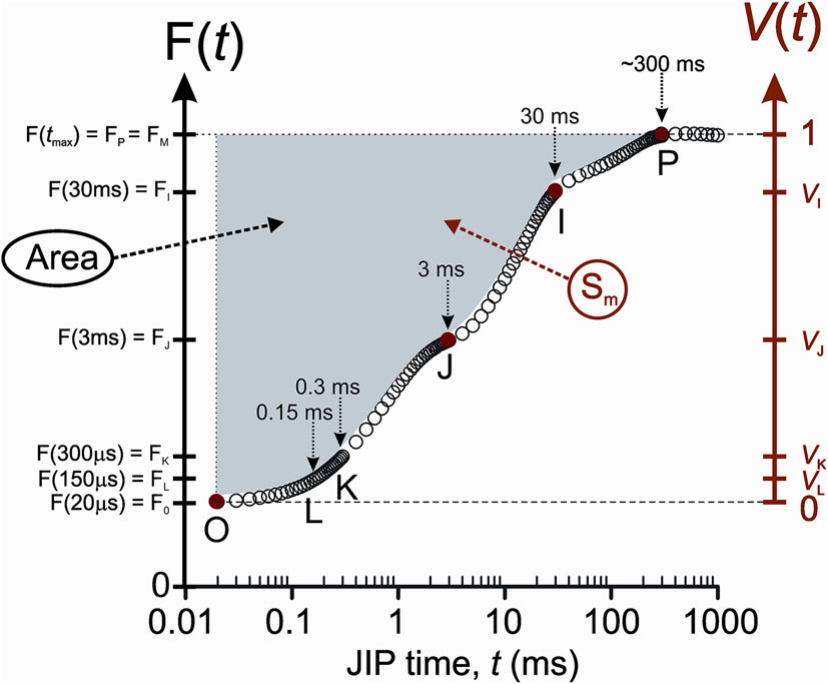
Time points and parameters used in the JIP test. On the left hand side, the unnormalized *F* scale associated with the complementary “Area” and on the right hand side, the *V* scale double normalized between O and P associated with the normalized area Sm (Goltsev, unpublished data)

The idea that the fluorescence rise OJIP contains a lot of information on the photosynthetic system is already quite old. OJIP transients have been compared to a bar code for photosynthesis (Tyystjärvi et al. 1999) and extensive attempts to simulate OJIP transients have been made (see Lazár and Schansker (2009) for a review of these efforts). In 1991, Strasser and Govindjee published an article on the recording of the full fluorescence rise kinetics OJIP between 40 μs and 1 s using a PEA instrument (see Strasser et al. 1995 for details). Four years later, Strasser and Strasser (1995) proposed a method to analyze these OJIP transients that was centered on the J-step [observed after 2–3 ms of strong illumination and equivalent to the *I*_1_ step of Schreiber (1986)], which they called the JIP test (see Fig. 7).

The theoretical basis of the JIP test has been described in detail by Strasser et al. (2004). In the JIP test, OJIP transients are used to make a flux analysis, i.e., an analysis of the fate of photons absorbed by the PSII antennae (trapping, forward electron transport beyond *Q*_A_ and dissipation as heat). In the JIP test, the J-step is taken as the border between single and multiple turnovers. If we define multiple turnovers here as events related to electron transport beyond PSII, then this claim still remains valid. The JIP test depends strongly on the assumption that the *F*_O_-to-*F*_M_ rise reflects the reduction of *Q*_A_. The concept is internally consistent but the theoretical foundation of the interpretation of the parameters disappears the moment that this assumption turns out to be wrong (see Schansker et al. 2011, 2014 for a discussion of this point). An alternative approach to the interpretation of the OJIP transients is a classical physiological characterization of the various features of the fluorescence rise.

In the JIP test, it is assumed that the relative position of the J-step between *F*_O_ and *F*_M_ (i.e., *V*_J_, giving rise to the JIP-parameter 1 − *V*_J_ or *Ψ*_O_) gives information on photosynthetic electron transport beyond *Q*_A_ (e.g., Strasser et al. 1995, 2004). A physiological characterization of this feature, on the other hand, suggests that the parameter *V*_J_ depends on the redox state of the PQ-pool in darkness (Tóth et al. 2007a) and, under certain stress conditions, may also be affected by other factors, possibly the extent of stacking of the thylakoid membranes. In this case, electron transport beyond *Q*_A_ means a slowdown of the re-oxidation of *Q*_A_^−^ as the PQ-pool becomes more reduced, and fewer PQ molecules are bound to the *Q*_B_-site. Changes in *Ψ*_O_ may certainly point to stress.

In the JIP test, the parameters *F*_O_ and *F*_M_ were suggested to be a measure for the absorption flux (i.e., the number of photons absorbed per unit of time) per cross section (Strasser et al. 1995, 2004). With respect to this interpretation, it may be noted that a characterization of the changes in the *F*_O_ and *F*_M_ levels as a function of the Chl content of leaves showed that they are nearly insensitive to changes in the leaf chlorophyll content as long as the antenna sizes of the RCs remain unaffected (Dinç et al. 2012). However, we note that this observation probably does not apply to dilute algal and thylakoid suspensions.

Malkin (1966) and Murata et al. (1966) showed that the complementary area between the fluorescence transient and *F*_M_ in the presence of DCMU is proportional to the population of reduced *Q*_A_ molecules. In the JIP test, this principle is extended to the situation in the absence of DCMU, where the area between the fluorescence transient and *F*_J_ is assumed to equate one charge separation in all RCs, i.e., one electron transported, to which the total area above the OJIP transient can be normalized (see e.g., Strasser et al. 2004). Schansker et al. (2011, 2014) support and explain the relationship between the area above the OJIP transients (see Fig. 7) and the number of electrons that must be transported through the ETC before *F*_M_ is reached.

In the JIP test, it is assumed that the slope taken between *F*_O_ and *F*_150 μs_ is sensitive to a phenomenon called “connectivity,” i.e., the energy transfer between the antennae of several PSII RCs, whereas the slope taken between *F*_O_ and *F*_300 μs_ is insensitive to connectivity (Strasser and Stirbet 2001; and see Stirbet 2013 for a more in-depth discussion of connectivity in the absence of PSII inhibitors like DCMU).

The performance index [PI(ABS)] was introduced as an attempt to catch three different aspects of the photosynthetic activity of PSII in a single parameter (see Clark et al. 2000 for an early application of this parameter). PI(ABS) is the product of a parameter sensitive to the effective antenna size, a parameter based on the primary quantum yield of PSII and a parameter sensitive to changes in the relative position of *F*_J_. It is defined as:

$${\text{PI(ABS)}} = \frac{{\frac{{F_{\text{V}} }}{{F_{\text{M}} }}\,V_{\text{J}} }}{{\frac{{4(F_{{270\;{{\mu s}}}} - F_{\text{O}} )}}{{F_{\text{M}} - F_{\text{O}} }}}}\,\,\,\,\frac{{\frac{{F_{\text{V}} }}{{F_{\text{M}} }}}}{{1 - \frac{{F_{\text{V}} }}{{F_{\text{M}} }}}}\,\,\,\,\frac{{1 - V_{\text{J}} }}{{V_{\text{J}} }}$$

with *V*_J_ = (*F*_J_ − *F*_O_)/F_M_ − *F*_O_). It is another JIP test parameter that has been shown to correlate with other stress parameters under a series of conditions (e.g., Clark et al. 2000; Misra et al. 2001a, b; Oukarroum et al. 2006). Physiological studies have further shown that the IP phase of the fluorescence rise is related to electron transport through PSI (Kautsky et al. 1960; Munday and Govindjee 1969; Schansker et al. 2005) and that the (relative) amplitude of the IP phase is linked to the PSI content of the leaf (Oukarroum et al. 2009; Ceppi et al. 2012). The JIP test approach remains a good and fast way to screen a large number of samples (Kalaji et al. 2011a, b). However, once parameters that correlate with certain features of a stress have been identified, it should not be blindly assumed that the interpretation of these parameters as given by the JIP test is correct (see also Stirbet and Govindjee 2011 for a discussion of this topic). In addition, it should be kept in mind that the JIP test depends strongly on normalizations which are very sensitive to the correctness of the determined *F*_O_ and *F*_M_ values. For example, in the case of heat stress, it is not easy to determine the *F*_O_ and *F*_M_ values correctly (see Tóth et al. 2007b).

## Question 20. What kind of values may one expect for particular fluorescence parameters?

The *F*_V_/*F*_M_ values of plant species average approximately 0.83–0.84 in C3 plants under optimal conditions (Björkman and Demmig 1987; Pfündel 1998) and 0.78 in C4 plants (Pfündel 1998). Somewhat higher values have been described in certain broadleaved species. Lower values, on the other hand, are common in algae and lichens (see Trissl and Wilhelm 1993 for a discussion of these values). Stress conditions (e.g., photoinhibition) can significantly reduce these values (e.g., Björkman and Demmig 1987; Van Wijk and Krause 1991; Tyystjärvi and Aro 1996).

Photochemical quenching qP, non-photochemical quenching defined as qN [= 1 − (*F*_M_′ − *F*_O_′)/(*F*_M_ − *F*_O_)], and the PSII operating efficiency in the light (*Φ*_PSII_) can vary between 0 and 1 (see Question 14 for definitions of qP and *Φ*_PSII_). The theoretical range for the values of the non-photochemical quenching parameter NPQ [= *F*_M_/*F*_M_′ − 1] is from zero to infinity, but in most cases, it gives values between 0 and approximately 10. However, NPQ values higher than 10 have been reported in bryophytes from sun-exposed habitats (Marschall and Proctor 2004; see Buschmann 1999 for a discussion and comparison of qN and NPQ). High *Φ*_PSII_ values indicate that a large proportion of the light absorbed by the chlorophylls of the PSII antenna is converted into photochemical energy. At its upper limit, *Φ*_PSII_ could reach a value of 1, which would mean that all absorbed energy is used for stable charge separations in PSIIs. From a practical point of view, this cannot be the case, due to the fundamental inefficiency of PSII (triplet formation, a small probability of fluorescence, and heat emission on each transfer of excitation energy between chlorophylls), and the contribution of fluorescence emitted by PSI has also an effect on the calculation (see Question 3). Therefore, *Φ*_PSII_ can vary between zero and the *F*_V_/*F*_M_ value, which in C3 plants is about 0.83–0.85, in C4 plants around 0.78 and in algae often below 0.7 (Pfündel 1998; Trissl and Wilhelm 1993). qP values near zero indicate that most of the PSII RCs are closed, and their *Q*_A_ is in the reduced state. Values near 1 indicate that *Q*_A_ is in the oxidized state, and almost all of the PSII centers are open for photochemistry. The non-photochemical quenching coefficients qN and NPQ are assumed to be zero in the dark-adapted state, because then *F*_V_′ = *F*_V_ and *F*_M_′ = *F*_M_. However, in some cases, positive values of these coefficients can also occur in darkness (see Question 17).

In higher plants, the induction kinetics of non-photochemical quenching triggered by high light usually have a typical time dependence: they increase during the first minute of illumination due to initiation of electron transport and ΔpH formation preceding the activation of ATP synthase (e.g., Nilkens et al. 2010) and decrease again once the Calvin–Benson cycle is activated. This quenching is sensitive to the balance between the electron transport rate and its associated proton transfer toward the thylakoid lumen on the one hand and the rate of ATP synthesis and the associated release of protons from the thylakoid lumen on the other hand. This form of quenching (corresponding to qE quenching, see Question 15) relaxes quickly as soon as electron transport stops, e.g., as soon as the light is turned off (see e.g., Nilkens et al. 2010). Other processes contributing to NPQ have slower induction kinetics (see Questions 2.3 and 15) whose induction (e.g., photoinhibition) depends as well on light intensity. Higher non-photochemical quenching values related to higher values of qE under steady state conditions suggest a stronger imbalance between photosynthetic electron transport and the utilization of NADPH (reflected by lower qP values) (see e.g., Walters and Horton 1993). Under continuous and/or extreme stress, non-photochemical quenching can attain low values. This may in part be due to a loss of RCs. Photoinhibited PSII RCs lose their variable fluorescence, and as a consequence, this variable fluorescence can then no longer be quenched, which means less NPQ (Schansker and Van Rensen 1999). Low values may also be caused by decreased rates of linear electron transport generating a smaller transthylakoid proton gradient or to an increased permeability of the membrane due to lipid peroxidation caused by oxygen radicals, which will also reduce the build up of a ΔpH over the membrane.

Deviations from the NPQ induction kinetics have been described in some green algae, where the NPQ induction capacity varies strongly depending on the species (see e.g., Bonente et al. 2008). For example, in *Ulva laetevirens*, NPQ was induced with an early peak within the first minute of exposure to high light, followed by a decrease and a subsequent rise (Bonente et al. 2008).

## Question 21. Which assumptions are made when interpreting fluorescence transient measurements?

Both the quenching analysis and the JIP test (see Questions 15 and 19 for a discussion) are based on assumptions that were commonly made in the 1990s (e.g., van Kooten and Snel 1990 for the quenching analysis, Strasser 1996 for the JIP test and see also Stirbet and Govindjee 2011 for a list of assumptions). The most important assumption is that the fluorescence increase from *F*_O_ to *F*_M_ reflects mainly the reduction of *Q*_A_. This idea was first put forward by Duysens and Sweers (1963). However, this assumption was challenged almost from the beginning (see e.g., Delosme 1967). Delosme (1967) proposed the existence of two processes determining the fluorescence rise. His suggestion that the redox state of the PQ-pool could play a role (Delosme 1971) led to the idea that the *Q*_B_-site occupancy state was the second factor (see Samson et al. 1999); an idea that was extended further by Schansker et al. (2011) who suggested that the *Q*_B_-site occupancy state controlled the re-oxidation rate of *Q*_A_^−^ and who proposed on the basis of this idea that in the presence of *Q*_A_^−^ further excitations could induce conformational changes in the PSII RCs which would then cause an increase of the fluorescence yield. Considering the occupancy state idea, Schreiber (2002) proposed that the thermal phase might be explained by a reduction of the inactive branch of PSII. Vredenberg and co-workers (Vredenberg 2000; Vredenberg et al. 2006) developed another interpretation model, in which, in addition to *Q*_A_^−^, the IP phase is determined by the electric field, and JI rise reflects an inactivation of PSII RCs (associated with proton transport over the membrane) in which Pheo^−^ can accumulate. These alternative interpretations were challenged by Stirbet and Govindjee (2012). The first assumption that the *F*_O_-to-*F*_M_ rise is a reflection of the reduction of *Q*_A_ implies that it should always be possible to reach *F*_M,_ since all *Q*_A_ can be reduced if the light intensity is high enough (i.e., when the excitation rate is much higher than re-oxidation rate of *Q*_A_^−^ by forward electron transport and/or the exchange of PQH_2_ for PQ at the *Q*_B_-site). However, Schreiber (1986), Samson and Bruce (1996) and Schansker et al. (2006, 2008) showed in several ways that this is not the case.

A second, related, assumption is that there are no changes in non-photochemical quenching during a saturating pulse. Finally, a third assumption is that the parameters *F*_V_/*F*_M_ and *Φ*PSII are measures of the PSII quantum yield and that *Φ*PSII can be used to calculate the photosynthetic electron transport rate. For *Φ*PSII, this assumption has been partially verified experimentally, showing under several conditions a linear correlation between the calculated photosynthetic electron transport rate and the CO_2_ assimilation rate (Genty et al. 1989; Krall and Edwards 1992 and see Questions 29 and 30). We note that the meaning of the parameter *F*_V_/*F*_M_ has not been derived experimentally but is based on an analysis of so-called competitive rate equations (fluorescence emission competes with other processes like heat emission and photosynthesis) for the *F*_O_ and *F*_M_ states (Kitajima and Butler 1975; Kramer et al. 2004). This analysis is correct as long as the fluorescence rise between *F*_O_ and *F*_M_ is determined by the reduction of *Q*_A_ only (see Schansker et al. 2014 for a discussion of this point).

## Question 22. Are there naturally occurring fluorescence quenchers other than *Q*_A_?

Another fluorescence quencher that has been described extensively is P680^+^ (Butler 1972; Zankel 1973; Shinkarev and Govindjee 1993; Steffen et al. 2005). The short lifetime of P680^+^ keeps the population of this quencher low under most conditions. Simulation work has shown that under high light conditions, the highest concentration should occur around the J-step (Lazár 2003), which was supported by experimental observations (Schansker et al. 2011). However, P680^+^ quenching does not affect the *F*_O_ and *F*_M_ levels. Oxidized PQ molecules can also quench fluorescence, but only in isolated thylakoids and in PSII-enriched membranes (Vernotte et al. 1979; Kurreck et al. 2000; Tóth et al. 2005a) and not in leaves (Tóth et al. 2005a). Other quenchers such as Car^+^ and Chl^+^ have been proposed and shown to play a role at temperatures below 100 K (Schweitzer and Brudvig 1997) in the case of Chl_Z_^+^, an accessory chlorophyll molecule in the RC of PSII, or to have a very short lifetime at room temperature (Steffen et al. 2001) in the case of Car^+^. Neither of these quenchers seems to play a role in the fluorescence measurements discussed in this paper.

## Question 23. What is the difference between fluorescence emission spectra recorded at 77 K and those recorded at room temperature?

**Fig. 8:**
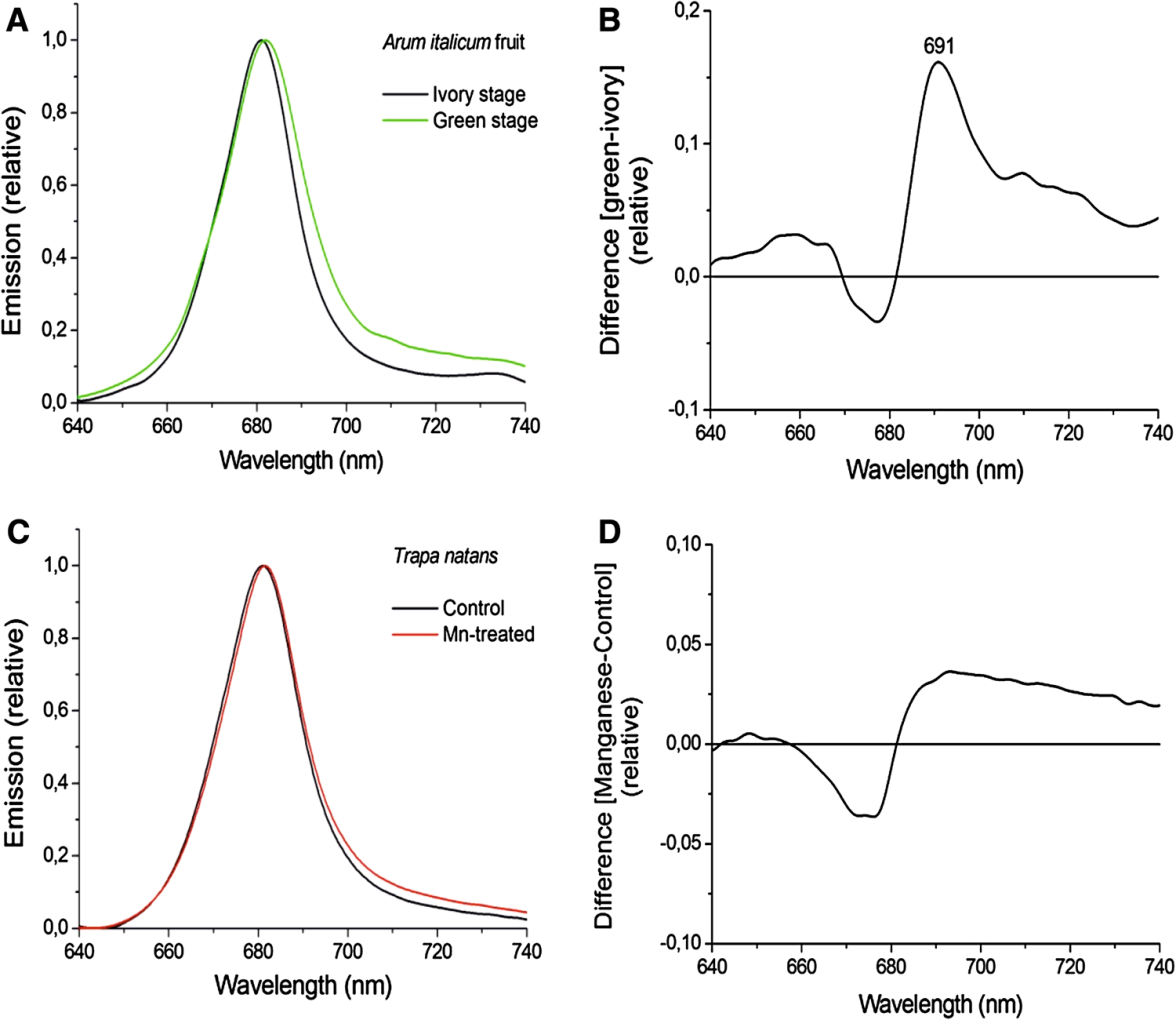
Examples of applications of room temperature (RT) fluorescence emission spectra. **a**, **b** RT spectra of two developmental stages of chloroplasts of the fruit of *Arum italicum*. In its early stage of development (ivory stage), the fruit contains a rudimentary thylakoid system in amyloplasts which upon maturation are converted to chloroplasts (green stage; see Bonora et al. 2000). A difference spectrum (normalized green stage—normalized ivory stage) **b** shows that a distinctive trait of the amyloplast-to-chloroplast transition is the gain in emission at around 691 nm, roughly corresponding to a PSII-core contribution. An in-depth analysis of spectra in this system showed that the F695/F680 fluorescence ratio undergoes changes parallel to *F*_V_/*F*_M_, assembly of LHCII-PSII supercomplexes, and carbon fixation (Ferroni et al. 2013). **c**, **d** RT spectra to improve the description of chloroplast responses to stress. In the example, spectra were recorded from leaves of the aquatic plant *Trapa natans*, which were treated or not with manganese. In this species, acclimation to manganese includes an accumulation of LHCII in the leaf chloroplasts (Baldisserotto et al. 2013). Increased RT emission at long wavelength, as shown in the difference spectrum (**d**), points to the occurrence in vivo of uncoupled aggregates of LHCII which contribute fluorescence at around 700 nm (Ferroni and Pancaldi, unpublished data)

In Question 2 Sect. 4, measurements of 77 K fluorescence emission spectra were introduced as a method to study PSII and PSI antennae. The recording of fluorescence emission spectra is much easier at room temperature. In this case, one dominant peak at ~684 nm is recorded, which is attributed principally to fluorescence emission by the PSII-core complex (including the core antennae CP47 and CP43) and further a shoulder at 710–740 nm corresponding to several fluorescence emission sources—particularly PSI-LHCI and several minor PSII bands (Fig. 8) (Franck et al. 2005; Krausz et al. 2005; Pancaldi et al. 2002). When the temperature is lowered, the 684 nm band is replaced by two bands, peaking at 685 and 695 nm, respectively; bands that in first instance were shown to be associated with the PSII core (Gasanov et al. 1979; Rijgersberg et al. 1979). The 695 nm band is due to fluorescence emission from CP47, whereas the 685 nm has been associated with fluorescence emission by CP43 [(Nakatani et al. 1984; for spectroscopic analyses of CP47 and CP43: see Alfonso et al. 1994 (for both); van Dorssen et al. 1987 (CP47); Groot et al. 1999 (CP43)]. Srivastava et al. (1999) showed with an experiment on greening of peas how the 695 nm band increases in intensity as the PSII antenna size increases. In other words, despite CP47 being the source of the 695 nm emission, it is sensitive to the number of LHCII subunits bound to PSII. The relationship between the antenna size of PSII and the amplitude of the 695 nm band is further strengthened by the observation that chloroplast samples frozen in the presence of a ΔpH show a quenching of the 695 nm band (Krause et al. 1983). Based on a comparative study of photosynthetic mutants of *Chlamydomonas reinhardtii*, a relationship between LHCII-PSII association and emission intensity at ~695 nm has also been proposed at room temperature (Ferroni et al. 2011). To detect fluorescence emitted by LHCII itself as an individual peak at 680 nm, it is necessary to freeze the sample further to 4 K (see Govindjee 1995). However, a more or less distinct shoulder at 680 nm is often reported also at 77 K and attributed to the free LHCII trimers not linked with PSII in a stable association (Hemelrijk et al. 1992; Siffel and Braunova 1999; van der Weij-de Wit et al. 2007; Pantaleoni et al. 2009; Ferroni et al. 2013). At room temperature, the emission region around 680 nm, never visible as an individual peak in the spectrum, was also assigned to a contribution by free LHCII (Ferroni et al. 2011). Strasser and Butler (1976) showed that the strong band at 730 nm at 77 K is in part caused by energy transfer from PSII to PSI. Weis (1985) demonstrated that the absorption of PSII fluorescence emission by PSI can be reduced considerably using diluted “leaf powder” instead of whole leaf fragments. When using liquid samples, such as microalgae suspensions or isolated thylakoids, the PSI re-absorption of emitted light can be reduced by an adequate dilution of the sample. The re-absorption phenomenon also affects room temperature spectra, resulting in a relative increase in the emission at 710–740 nm and in a red shift of PSII emission (Franck et al. 2002).

Room temperature fluorescence emission spectra are not frequently used for photosynthesis studies, because the spectral components are not as well characterized as the 77 K spectra are (Franck et al. 2002; Ferroni et al. 2011). However, methods have been developed to resolve at room temperature the contribution of PSII and PSI to Chl *a* fluorescence under *F*_O_, *F*_M_, and steady state conditions (*F*_t_) (Franck et al. 2002, 2005). Figure 8 gives examples of two such applications. Room temperature fluorescence spectra have also been used to evaluate the response of photosynthetic organisms (microalgae and in higher plants) to some environmental stresses (Romanowska-Duda et al. 2005, 2010; Ferroni et al. 2007; Baldisserotto et al. 2010, 2012; Burling et al. 2011; Hunsche et al. 2011). Finally, such spectra have been used as well to characterize developmental aspects of the photosynthetic membrane (Pancaldi et al. 2002; Baldisserotto et al. 2005; Ferroni et al. 2009, 2013) and, as discussed in Question 25, to estimate leaf chlorophyll content.

## Question 24. Are the fluorescence rise kinetics sensitive to the chlorophyll content of the leaf?

For dilute solutions of chlorophyll molecules, the measured fluorescence intensity is proportional to the quantum yield of fluorescence multiplied by the number of photons absorbed and the chlorophyll concentration (Lakowicz 2009). On this basis, one would expect that the fluorescence intensity emitted by a leaf depends on the chlorophyll content of that leaf. However, as described under Question 4, the leaf is complex in optical terms, and it is difficult to predict if this physical law is really critical in determining the relationship between the chlorophyll content of the leaf and the fluorescence emission. Several experimental studies have addressed this question. Hsu and Leu (2003) showed that two leaves placed on top of each other emitted more Chl *a* fluorescence than a single leaf. However, this is a quite artificial construct, and it can easily be shown that the outcome of the experiment strongly depends on the way the leaves were oriented (e.g., both adaxial sides up, or adaxial side up for the top leaf and the abaxial side for the bottom leaf) (Ceppi and Schansker, unpublished observations, 2008). Sušila et al. (2004) attempted to show an effect of chlorophyll content using thylakoid suspensions differing in their chlorophyll content. Thylakoid suspensions are homogeneous in their properties, whereas under natural conditions, a change in the chlorophyll content will be accompanied by an adaptation (change in antenna sizes and/or changes in PSI:PSII ratio) of the individual chloroplasts inside the leaf to their new light environment (see Question 4). To address the effect of changes in the chlorophyll content of a leaf on the measured fluorescence properties, it is important to find a natural system in which the leaves can acclimate to the effects of the changing chlorophyll content. Sugar beet plants grown hydroponically in the absence of magnesium or low sulfate concentrations show a gradual loss of chlorophyll; the activity of the remaining ETCs remains largely unaffected, and there were no overall changes in the antenna size (effect on Chl *a*/*b* ratio was small). Under these conditions, an up to fivefold decrease in the chlorophyll content left the *F*_O_ and *F*_M_ values unchanged and had only a marginal effect on the fluorescence rise kinetics (Dinç et al. 2012). On the other hand, changes in the PSII antenna size did have an effect on the *F*_M_-intensity (Dinç et al. 2012). In conclusion, there is little indication that a stress-induced Chl loss in leaves would complicate the interpretation of Chl *a* fluorescence measurements.

## Question 25. Can the leaf chlorophyll content be measured using fluorescence?

Chlorophyll fluorescence emission spectra can be used to determine the chlorophyll content of green plants (Buschmann 2007). The ratio between chlorophyll fluorescence at 735 nm and that at 700 nm (F735/F700) is linearly proportional to chlorophyll content (Gitelson et al. 1999). Conversely, as discussed in Question 24, the *F*_M_ and *F*_O_ values are not related to the chlorophyll content in leaves (Dinç et al. 2012). It may also be noted that there are simple chlorophyll meters on the market (CL-01, Hansatech Instruments, UK; SPAD meter, Minolta, Japan; CCM-200, Opti-Sciences, USA) that can be used to follow changes in the leaf chlorophyll content (see e.g., Cassol et al. 2008; Dinç et al. 2012). These measurements can then be calibrated against measurements of the chlorophyll extracted from leaf areas measured before with the chlorophyll meter (see e.g., Dinç et al. 2012). Chl measurements on dark-adapted leaves seem to give more reproducible results than measurements made on light-adapted leaves (Ceppi and Schansker, unpublished data, 2008). If the chlorophyll meter is used over the day on the same leaf, the readings change (Mishra, unpublished data, 2010), e.g., due to chloroplast movements, which change the absorbance properties of the leaf (see Wada 2013 for a review on chloroplast movements). Chloroplasts are known to re-arrange themselves inside the cell in response to the ambient blue light intensity, adapting the absorbance properties of the leaf to the circumstances (Sakai et al. 2001; Kasahara et al. 2002). This does not only affect chlorophyll meter measurements, but also normal fluorescence measurements (Brugnoli and Björkman 1992).

In practice, values measured using a Chl meter are often used as indicators for relative Chl changes. In that case, we assume that the measured values are a linear function of the leaf chlorophyll content between zero and the value measured on control leaves. However, in that case, it is important to test the validity of this assumption for each plant species and for each stress studied (Mishra, unpublished data, 2013).

## Question 26. Is it possible to compare different leaves?

It is easy to take randomly two leaves from two plants of the same species and to make a fluorescence measurement. But is it truly possible to compare these two measurements? It is likely that a difference in maximum fluorescence amplitude will be observed. Especially, when studying OJIP transients, the kinetics are often more interesting than the absolute amplitude, and in that case, the difference in the fluorescence amplitude is eliminated by double normalization between *F*_O_ and *F*_M_. Arithmetically, this is done in the following way: (*F*_t_ − F_O_)/(*F*_M_ − *F*_O_). The effect of this calculation is to rescale each fluorescence value in a range going from 0 (corresponding to *F*_O_) to 1 (corresponding to *F*_M_). For a comparison of the kinetics of the individual rise phases of the OJIP transient, the same approach can be used. To compare the kinetics of the OJ-rise, the measured transient can be double normalized between O and J [i.e., (*F*_t_ − *F*_O_)/(*F*_J_ − *F*_O_)]. In terms of nomenclature, double normalizations turn *F* values into so-called *V* values, like *V*_J_, which is the double normalized *F*_J_ value (see Strasser et al. 2004).

An important source of variability between leaves is the development of stress symptoms. A common stress-related effect is chlorosis, and it has been argued that a change in the chlorophyll content of the leaf has an impact on the fluorescence kinetics and thereby invalidates the analysis (Hsu and Leu 2003; Susila et al. 2004) but as discussed in Question 24, this is not the case as long as chloroplasts can adapt to their new light environment. In addition, if the development of the stress effects is followed over time, the gradually changing fluorescence properties will help the interpretation of the data.

A comparison of leaf fluorescence measurements on stressed and unstressed plants in the field is hampered by the fact that such leaves are often acclimated to completely different light environments. It is important to realize that growth light intensity affects the stoichiometries and composition of many components of the photosynthetic membrane like the PSII to PSI ratio, the LHCII to PSII ratio, and the amount of PSII-LHCII supercomplexes (e.g., Leong and Anderson 1984a, b; Walters and Horton 1994; Dietzel et al. 2008; Wientjes et al. 2013). Therefore, it is of fundamental importance that the light environment (full sunlight, shade, deep shade) of leaves/plants to be compared has been adequately analyzed before the effect of a certain stress is addressed by fluorimetric techniques. Several papers illustrate this, e.g., stressed and unstressed plants were compared by van Heerden et al. (2007), whereas Zubek et al. (2009) compared leaves of plants with and without mycorrhiza, both ascribing the observed difference in the initial slope of the measured OJIP transients to an effect on the oxygen evolving complex of PSII. An alternative and more likely explanation—a difference in the effective antenna size between the samples due to differences in the growth light conditions—was not considered.

In summary, comparing leaves that develop under similar light conditions is relatively easy; however, comparing leaves that were growing under different light regimes is fraught with complications and should be avoided.

## Question 27. Can measurements made with different instruments during a large-scale field survey be compared in absolute terms?

It is important to be aware that the use of different instruments, even from the same company and the same type, may yield different results in absolute terms. The light source used for saturating pulses of modulated instruments may age over time reducing its light intensity. The strength of the red LEDs of HandyPEAs often differs between instruments. When comparing measurements made with different types of instruments, differences may also be due to the specific geometry of the measuring cell or to the use of light sources emitting at different wavelengths. It is possible to reduce these differences by determining the light intensity dependence of the parameters of interest and using these data to change settings in order to obtain comparable results. Differences in wavelengths of the exciting light may be impossible to correct for. Green light for example has been shown to probe deeper in the leaves than red light; blue light is even more efficiently absorbed than red light (Terashima et al. 2009).

An example of the phenomenon, described above, is a study in which the same leaves were measured with different HandyPEA instruments (Bussotti et al. 2011a) calibrated with identical settings (lamp intensity = 3,000 μmol photons m^−2^ s^−1^, time = 1 s, gain = 1). Both original and normalized transient curves were compared. Original curves differed consistently (both the extreme values of *F*_O_ and *F*_M_ showed a large range of variability), but the differences decreased consistently after normalization (double normalization between *F*_O_ and *F*_M_—see Question 26 for a definition). The parameter *F*_O_/*F*_M_ (parameter which is sensitive to changes in heat dissipation in the PSII antenna), as well as the normalized steps of OJIP transients—J and I (fluorescence intensities at 2–3 and 30 ms, respectively)—showed very little variability when comparing the measurements of the different instruments with a coefficient of variation (CV = SD/Mean) ranging from 3 to 5 %. The parameter PIabs, which consists of the product of a parameter sensitive to the effective antenna size, a parameter based on the maximum quantum yield of PSII, and a parameter sensitive to changes in the relative position of *F*_J_ (see Question 19) showed a very high variability among instruments (PIabs showed a CV = 30 %; Bussotti et al. 2011a). The high intrinsic variability of PIabs between instruments is due to the fact that this parameter is sensitive to the initial slope of the fluorescence rise and the relative position of the J-step, two factors that are both relatively sensitive to the light intensity of the beam. This high intrinsic variability makes the PIabs less useful for large, multi-instrument surveys.

In conclusion, in the case of small-scale experiments, it is always preferable to use the same instrument for all the measurements of an experiment.

## Question 28. How should a sampling campaign be organized for an ecosystem?

Large-scale surveys should be carried out using a robust sampling design. Criteria and examples of such designs can be found in many statistical manuals and textbooks (see Elzinga et al. 2001). Here, we discuss some specific issues related to the assessment of fluorescence parameters.

Two problems widely discussed in the context of forest health monitoring (Luyssaert et al. 2002) and other ecosystems (Tuba et al. 2010) are intercalibration and harmonization. Here, “intercalibration” refers to procedures aimed at reducing the differences between instruments discussed in Question 27, and “harmonization” refers to the sampling strategy. The main issues are the variability of the leaf responses within the crown/canopy and the ecological scale of the investigation (assessment of the response of the whole tree/plant, or of a target population of leaves).

A complete representation of a plant should take into account the different levels, age, and position of leaves. This would be the approach of choice but would require a large number of samples, and this would be difficult to realize in large-scale sampling. Thus, normally only one or a few leaf positions (e.g., sun leaves in the upper part of the crown, south exposed leaves, flag leaves, or fully developed leaves) are considered, depending on the purpose of the survey.

The number of leaves to be sampled depends on the internal variability of the parameters of interest. The following formula can be used for this calculation:

$$ n \, = \, Z_{\alpha }^{2} s^{2} / \, B^{2} $$

where *n* is the sample size; *Z*_α_ is the standard normal coefficient (= 1.96 for a 95 % confidence level); *s* is the SD; *B* is the desired precision level expressed as percent of the mean value (Elzinga et al. 2001; Gottardini et al. 2014). A recent study of boreal forests (Pollastrini et al. 2014) found that, in the higher external part of a crown of *Betula pendula*, the CV among different leaves was very low for *F*_V_/*F*_M_ (1.6 %), and increased for the parameters related to the step J (1 − *V*_J_, CV = 7 %) and the step I (Δ*V*_IP_ = 1 − *V*_I_, CV = 14 %). We mention here that this type of studies demonstrated that the IP phase, linked to the PSI content (Oukarroum et al. 2009; Ceppi et al. 2012), is quite sensitive to different types of stress; e.g., it decreased in response to ozone (Bussotti et al. 2011b) and nitrogen deprivation (Nikiforou and Manetas 2011), while it increased in response to high light conditions (Desotgiu et al. 2012).

In order to sample as many leaves as possible during a single day, sampling must be performed during the whole day and cannot be limited to specific hours. As a consequence, leaves are sampled under different conditions of short-term light acclimation and different extents of photoinhibition. To reduce the associated variability, it is necessary to allow the regulatory mechanisms induced by the ambient light to relax and to allow the leaves to recover from photoinhibition, which means a sufficient period of at least 4–5 h of dark acclimation at a constant temperature must be made before measurement. In addition, to avoid the onset of leaf senescence or the induction of other stress factors that can change the physiological state of the leaf during sampling and dark acclimation of the leaves, all fieldwork must be performed as fast as possible. Managing a large number of samples in a short time, e.g., 1,000 samples in one day, requires fast instruments/experimental protocols. OJIP transients need less than 1 s of illumination, and their analysis is best suited for this kind of application.

## Question 29. What additional information can be obtained from simultaneous measurements of CO_2_ exchange and chlorophyll fluorescence?

Modern Infrared gas analyzers (IRGAs; such as the CIRAS-3, PP Systems and LI-COR 6400) allow gas exchange and fluorescence to be measured simultaneously. This combination can provide information about effects on the photosynthetic ETC, Calvin–Benson cycle activity, and diffusional limitations at the same time. Additionally, it is possible to determine chlorophyll fluorescence parameters under particular conditions (e.g., increasing CO_2_ concentrations or low O_2_ concentrations) to determine the maximum electron transport rate. In this way, effects of a certain treatment can be more precisely assigned to a particular process in the whole photosynthetic apparatus than the use of these techniques individually would allow (see e.g., Laisk and Loreto 1996; Laisk et al. 2005).

Three potential applications for simultaneous measurements have been proposed in the literature:

(i) *Analysis of alternative sinks of electrons* (e.g., Flexas et al. 1998; Bota et al. 2004). Discrepancies between the electron transport rate (ETR) and the net CO_2_ assimilation rate (*A*_n_) are an indicator of the existence of alternative electron sinks. For example, an increased ETR/*A*_n_ ratio indicates the existence of other electron sinks (e.g., Mehler reaction, photorespiration, nitrate reduction) in competition with CO_2_ assimilation (e.g., Bota et al. 2004). An important cause for an increase in ETR/*A*_n_ is photorespiration (e.g., Galmés et al. 2007). Comparing measurements made at 2 % O_2_ (suppression of photorespiration) with measurements made at 21 % O_2_ (ambient) allows a quantification of this process (Rosenqvist and van Kooten 2003).
(ii) *Calculation of CO*_*2*_ *diffusion resistance/conductance in the mesophyll, which in bifacial leaves is formed by the palisade and spongiform tissues* (von Caemmerer 2000). Mesophyll conductance is an important variable controlling CO_2_ diffusion to the carboxylation site of Rubisco. Several methods have been proposed to estimate mesophyll conductance in leaves (for a detailed description of these methods, see e.g., Warren 2006; Flexas et. al. 2008). One of these methods is based on IRGA measurements (measurements of CO_2_ assimilation, *A*_n_/*C*_i_ curves) and the electron transport rate from chlorophyll fluorescence (e.g., Flexas et al. 2006)—a detailed description of this method is available elsewhere (Loreto et al. 1992; Evans and Loreto 2000; Flexas et al. 2008).
(iii) *Sink limitations in photosynthesis* (Rosenqvist and van Kooten 2003). In a variation of point (i) above, simultaneous IRGA and chlorophyll fluorescence measurements made at low (2 % O_2_, which suppresses photorespiration in C3 plants), and ambient (21 % O_2_) oxygen concentrations can be used to estimate changes in source–sink relationships in leaves (Rosenqvist and van Kooten 2003). Under non-sink restrictions and 2 % oxygen, the CO_2_ assimilation rate (*A*_n_) should increase, and the ETR should remain the same. By contrast, if the leaf is sink-limited, lowering the oxygen concentration to 2 % will not affect *A*_n_, whereas the ETR will decrease (down-regulation by final product).

## Question 30. Can the wavelength dependence of the quantum yield for CO_2_ fixation be predicted by measuring chlorophyll fluorescence?

Emerson and Lewis (1943) observed that the quantum yield for O_2_ evolution is wavelength dependent and that it dropped off quickly at wavelengths longer than 700 nm. Similar wavelength dependence is observed for *Φ*co_2_ (McCree 1972; Inada 1976; Hogewoning et al. 2012). Typically, photosynthetic rates are higher when a leaf is illuminated with light in the red region (600–680 nm), compared with an equal number of photons in the blue or the green regions of the light spectrum. Beyond 700 nm (i.e., the FR region), *Φ*co_2_ declines rapidly to nearly zero at about 730 nm.

Genty et al. (1989) demonstrated that the PSII operating efficiency (i.e., *F*_q_′/*F*_M_′ or *Φ*_PSII_) correlates linearly with *Φ*co_2_ if the photosynthetic steady state is induced by white light of different intensities, while photorespiratory activity is low. This is always the case in C4 plants and in C3 plants, this occurs when the O_2_ concentration is low (1–2 %) (see also Question 29; Genty et al. 1989; Krall and Edwards 1992). In contrast to the relationship between *Φ*co_2_ and light intensity, Chl *a* fluorescence measurements are unsuitable for the estimation of the relationship between *Φ*co_2_ and the wavelength of irradiance used. To understand why, it is important to consider the factors that may affect the wavelength dependence of both *Φ*co_2_ and *Φ*_PSII_.

First, different wavelengths are not reflected and transmitted to the same extent by leaves. Hence, the fraction of light absorbed by a leaf is wavelength dependent (e.g., Vogelmann and Han 2000; see also Question 4). This also explains why most leaves are green and not, for example, black—relatively more green light is reflected and transmitted than red and blue light, and therefore, the fraction of red and blue light absorbed by a leaf is higher than the fraction of green light that is absorbed (Terashima et al. 2009). A lower fraction of incident light reaching the photosystems will directly result in a loss of *Φ*co_2_ on an incident light basis. However, at low light intensities in the linear part of the light-response curve, there are no limitations for the electron flow on the acceptor side of PSII. Therefore, within a range of low light intensities (typically between PPFD of 0 and 50 µmol photons m^−2^ s^−1^, or an even narrower range for shade-leaves), *Φ*_PSII_ does not necessarily change as a result of small changes in the light intensity. Beyond this range of low light intensities, *Φ*_PSII_ decreases when the light intensity increases, due to limitations for the electron flow on the acceptor side of PSII (see Question 2 Sect. 1 for electron transfer rates on the acceptor side of PSII). Thus, wavelength-dependent differences in the fraction of incident light reaching the photosystems are reflected by differences in *Φ*co_2_, but at low light intensities not necessarily by differences in *Φ*_PSII_.

Second, carotenoids differ in the efficiency (35–90 %) with which they transfer excitation energy to chlorophylls, whereas the chlorophyll to chlorophyll energy transfer efficiency in antenna complexes is nearly 100 % (Croce et al. 2001; de Weerd et al. 2003a, b; Caffarri et al. 2007). The transfer efficiency of carotenoids depends on their chemical structure and position within the photosynthetic apparatus. Carotenoids have absorption maxima in the blue and green regions, and therefore, blue light is used less efficiently by the photosystems than e.g., red light. Wavelength-dependent differences in the fraction of light absorbed by carotenoids affect the fraction of absorbed light reaching the RCs of the photosystems. This leads to the same argument as in the previous paragraph, i.e., this effect decreases *Φ*co_2_ but at low light intensities does not necessarily decrease *Φ*_PSII_.

Third, leaves contain non-photosynthetic pigments such as flavonoids and free carotenoids. These pigments predominantly absorb light in the UV region but also in the blue and green part of the spectrum. These non-photosynthetic pigments are not connected to the photosystems and do not transfer the absorbed energy to the photosynthetic apparatus (see Question 31 for a discussion of these compounds and their detection). The absorption of light by non-photosynthetic pigments will reduce the fraction of the incident light reaching the photosystems especially in the blue and to a smaller extent in the green. Again this will affect *Φ*co_2_ at these wavelengths but at low light intensities not necessarily *Φ*_PSII_.

Finally, the pigment composition and absorbance properties of PSI and PSII differ, and therefore, the balance of excitation between the two photosystems is wavelength dependent for a given state of the photosynthetic apparatus (e.g., Evans 1986; Chow et al. 1990a, b; Melis 1991; Walters and Horton 1995; Hogewoning et al. 2012). In practice, when light within a narrow-band wavelength range is used to illuminate a white-light acclimated leaf, one of the two photosystems is often excited more strongly than the other. Any imbalance in excitation between the two photosystems results in a loss of *Φ*co_2_. This wavelength dependence is especially clear in the FR region. FR light still quite efficiently excites PSI but is very inefficiently absorbed by PSII (see Question 16). This is called “the red drop” and, as noted above, this leads to a rapid decline of *Φ*O_2_ and consequently of *Φ*co_2_ as well at wavelengths longer than 685 nm. Obviously, when PSI is excited strongly by FR light, but PSII is excited only very weakly, electron flow from PSII to PSI is not restricted, and therefore, *Φ*_PSII_ will be high. However, due to the inefficient absorption of the FR photons by PSII, linear electron flow is low, and therefore, *Φ*co_2_ is low for FR light. On the other hand, if PSII is excited more strongly than PSI, the consequent loss of *Φ*_PSII_ is reflected by a proportional loss of *Φ*co_2_. Wavelengths in the range around 480 nm (blue) result in the strongest preferential excitation of PSII and therefore the strongest loss of both *Φ*co_2_ and *Φ*_PSII_ (Hogewoning et al. 2012). However, *Φ*_PSII_ is also an unreliable measure of *Φ*co_2_ for these blue wavelengths, due to the absorption by carotenoids and non-photosynthetic pigments (see above).

In summary, *Φ*_PSII_ calculated from chlorophyll *a* fluorescence measurements is an unsuitable parameter for estimating the wavelength dependence of *Φ*co_2_. Wavelength-dependent changes in (1) the absorbed light fraction, (2) the light fraction absorbed by photosynthetic carotenoids, and (3) the light fraction absorbed by non-photosynthetic pigments, directly affect the fraction of photons reaching the photosystems and therefore *Φ*co_2_. However, at low light intensities, changes in the fraction of photons reaching the photosystems may not affect *Φ*_PSII_. Furthermore, (4) some wavelengths preferentially excite PSI, resulting in high *Φ*_PSII_ values but low *Φ*co_2_ values. As a consequence, for a reliable measurement of the wavelength dependence of *Φ*co_2_, gas exchange measurements remain the gold standard.

## Question 31. Can anthocyanins and flavonols be detected by chlorophyll fluorescence?

In vivo non-destructive determination of anthocyanins and flavonols in green parts of plants can be made using the fluorescence excitation ratio method (FER) (Bilger et al. 1997; Agati et al. 2011). The FER method is based on the measurement of chlorophyll fluorescence induced by different excitation wavelengths. The extent of absorbance of light by the epidermal polyphenols can be derived on the basis of the ratio of chlorophyll fluorescence emission intensities induced by a standard red beam and a UV–VIS beam (wavelengths strongly absorbed by epidermal polyphenols). The role of different anthocyanins and flavonols can be distinguished by choosing appropriate wavelengths based on the specific absorbance spectra of the different anthocyanins and flavonols.

The chlorophyll fluorescence excitation technique was originally developed to assess UV-absorbing compounds in the leaf epidermis (Bilger et al. 1997). Ounis et al. (2001) extended the method developing remote sensing equipment (dual excitation FLIDAR) to study polyphenols not only in leaves but also in canopies of trees. This method has also been used for the determination of the presence of flavonoids, including anthocyanins, in the skins of fruits like grapes (Kolb at al. 2003), apples (Hagen et al. 2006), and olives (Agati et al. 2005). Betemps et al. (2011) showed that in fruits, the anthocyanins and other flavonoids localized in the outer skin layers reduce the chlorophyll fluorescence signal in proportion to the concentration of these polyphenols.

Pfündel et al. (2007) investigated two different types of commercial portable UV fluorometers for in vivo screening of anthocyanins and carotenoids in leaves. The UV-A-PAM fluorometer (Walz, Germany) makes use of a blue reference beam, whereas the Dualex fluorometer (FORCE-A, France) makes use of a red reference beam. For measurements on green leaves, the two instruments gave similar results, whereas the anthocyanins common in fruits absorbed part of the blue light of the UV-A-PAM reference beam which led, for fruits, to higher estimates for epidermal UV transmittance compared to that by the Dualex fluorometer. Pfündel et al. (2007) also noted that the absence of Chl *b* (e.g., in the barley chlorina f2 mutant) affected the determination of the polyphenols. Ben Ghozlen et al. (2010) developed and described an improved instrument, which they called the Multiplex (FORCE-A, France). It contains four light-emitting diodes (LEDs): UV-A (370 nm), blue (460 nm), green (515 nm), and red (637 nm) and three diodes to detect fluorescence emission at 590, 685, and 735 nm. The three diodes allow corrections for differences in the chlorophyll content of the sample. The red LED provides the reference beam, because it corresponds to a wavelength not absorbed by anthocyanins or flavonols. The fluorescence induced at this wavelength is compared with the fluorescence intensity induced by the excitation wavelength specific for the polyphenol of interest (e.g., green 515 nm light for anthocyanins or 370 nm UV-A light for flavonols). Ben Ghozlen et al. (2010) derived formulas to correlate these ratios with the actual polyphenol content of the sample.

In summary, a fluorescence-based method and accompanying equipment have been developed to determine the anthocyanin and flavonol content of leaves and fruits. In the case of fruits, the choice of the color (blue or red) of the reference beam influences the results, something that does not affect leaf measurements.

## Question 32. Can Chl *a* fluorescence be used as an indicator for a specific stress in plants?

To use Chl *a* fluorescence as a tool to identify a specific stress, the effects of that stress on the photosynthetic apparatus must be understood (Kalaji et al. 2012a, b). If heat stress destroys the donor side of part of the PSII RCs, it reduces the electron donation capacity of all PSII RCs together and, as a consequence, causes a slow down of the JI rise as measured by a PEA-type instrument (Srivastava et al. 1997 and see also Schreiber and Neubauer 1987). It also changes the recombination properties of the affected PSII RCs when measuring DF (Čajánek et al. 1998). In extreme cases, when all or nearly all PSII donor sides have been destroyed, the fluorescence rise levels off after ~300 μs of illumination (i.e., one charge separation) and then declines; this fluorescence pattern is called the K-peak (Guissé et al. 1995; Srivastava et al. 1997; Lazár et al. 1997). UV radiation may also destroy the donor side of PSII (e.g., Ohnishi et al. 2005; Hakala et al. 2005), but, at the same time, may have additional affects on the PSII RC (e.g., Vass et al. 1996) and, thereby, on the fluorescence kinetics. For both drought stress and sulfate deficiency, it was shown that they affect PSI (Oukarroum et al. 2009; Ceppi et al. 2012). Again, a combination of experimental phenomena is needed to distinguish these stress conditions. Another complication is that the PSII to PSI ratio that affects the parameter Δ*V*_IP_ is regulated by the growth light intensity and quality as well (Leong and Anderson 1984b; Lee and Whitmarsh 1989; Chow et al. 1990a, b). Finally, there are considerable kinetic differences between the OJIP transients obtained from different plant species (Kirova et al. 2009). This means that good references are needed to determine if something is a stress effect, taking into account the normal plasticity of the OJIP transients. The available physiological studies often concentrate on the effects of severe stress under laboratory conditions. In the field, milder stress effects are often observed, which possibly have to be distinguished from other sources of variability, so that additional research efforts will be needed to obtain reliable “fingerprints” for a particular stress. An example of the type of research needed is a study by Kalaji (2011) who characterized the effects of 16 abiotic stresses on the fluorescence properties of two Syrian landraces (cvs. Arabi Abiad and Arabi Aswad) of barley (see also Kalaji and Guo 2008).

Another approach is to make mathematical analyses of sets of OJIP transients in combination with DF and 820 nm transmission transients. Goltsev et al. (2012) trained an artificial neural network to estimate the relative water content (RWC) of leaves; they obtained a correlation value of *R* ^2^ = 0.98 between the estimated RWC value and the gravimetrically determined RWC value of the analyzed leaves.

In France, commercial software was developed that compares measured OJIP transients with a database of fluorescence transients measured on plants of dozens of genotypes of agricultural and horticultural crops suffering from deficiencies of the following elements: N, Fe, Mn, Mg, P, S, Ca, and B. This approach has similarities with the one discussed above, but it is more ambitious in its scope. This software is at the moment very popular among farmers, especially in Poland, Ukraine, and Russia, where it is promoted by producers of fertilizer. Kalaji et al. (unpublished data, 2013) did many experiments to test the software and suggested analysis, comparing the fluorescence analysis with the chemical analysis of several plant species grown under different conditions of nutrient deficiency. These studies suggested that this method needs further improvements to achieve a general validity.

For the moment, it is not possible to identify specific stresses using Chl *a* fluorescence. As noted above, different stresses may have similar effects on the photosynthetic system. In addition, in the field, plants are often subjected to several stresses at the same time, e.g., a combination of drought, high light, and heat stress. In the laboratory, it is possible to induce clear symptoms, whereas in the field, a combination of a less severe stress and acclimation may cause less specific symptoms. In other words, the complicated relationship between fluorescence kinetics, stress, and natural variation is not yet sufficiently well understood to use fluorescence measurements as fingerprints for specific stresses under natural conditions.

## Question 33. Is Chl *a* fluorescence a useful tool for the monitoring of aquatic ecosystems?

The use of Chl *a* fluorescence measurements for the study of aquatic environments is a topic by itself, and here only a few points are made. This topic was reviewed in depth in a recent book edited by Suggett et al. (2011).

The estimation of biomass production in aquatic environments is one of the research topics in which fluorescence techniques have played a major role and for which special equipment was developed. Falkowski and Kolber (1990) developed a submersible pump-probe instrument (see Question 2 Sect. 1 for the principle) to study biomass productivity profiles along the water column in the ocean. Further, Kolber et al. (1998) discussed a new fluorescence approach, which they called the FRR approach which was originally developed for aquatic studies. Instead of continuous light, subsaturating excitation flashes (of which the spacing can be varied) are used to induce photosynthesis. With these flashlets, the authors could create STFs as well as multiple turnover pulses and, at the same time, study the dark relaxation kinetics of fluorescence. One of the parameters that could be determined was the effective PSII antenna cross section. Using a Xenon-PAM (Walz, Germany), Geel et al. (1997) studied several classes of aquatic organisms in order to derive the oxygen evolution activity of these organisms on the basis of fluorescence measurements. Kromkamp and Forster (2003) have reviewed such studies.

Another important difference between measurements on plants and measurements in an aquatic environment is that aquatic samples often consist of a mixture of photosynthetic organisms. To cope with this problem, several instruments were developed that make use of differences in the pigment composition of different classes of photosynthetic organisms. Schreiber (1998) has described an instrument built by Kolbowski and Schreiber called the PHYTO-PAM Phytoplankton analyzer (Walz, Germany). The instrument does not use a monochromatic modulated beam but excites the samples alternately with weak 10 μs light pulses of 470, 535, 620, and 650 nm (inducing *F*_O_) to distinguish between cyanobacteria, green algae, and diatoms. Deconvolution of the algal composition was possible using reference spectra derived from pure cultures of particular classes of organisms. In addition, the instrument allowed the estimation of the activity of these classes of organisms using saturating light pulses (see Questions 2.3, 10, and 15).

Beutler et al. (2002) built a submergible instrument called bbe Fluoroprobe^TM^ (Moldaenke, Germany) that made use of five excitation wavelengths (450, 525, 570, 590, and 610 nm) with which particular accessory pigments can be relatively specifically excited allowing the detection of peridinin containing dinoflagellates and Pyrrophyta, chlorophyll *b* containing green algae, fucoxanthin containing diatoms, and zeaxanthin as well as phycobiliprotein containing cyanobacteria or cryptophycaea. Reference spectra were used to determine the chlorophyll content associated with each class. Rolland et al. (2010) using this equipment for a monitoring study of the Marne reservoir summarize its application in monitoring studies up till that time and note that it can be used down to 100 m, and that it has a short response time.

Further, Schreiber et al. (2012) have developed a new Multi-Color-PAM (Walz, Germany) instrument that combines multi-spectral excitation (400, 440, 480, 540, 590, and 625 nm) with the possibility to measure fast fluorescence kinetics as well as the absorption cross section of PSII antennae.

Photosynthetic aquatic organisms (including aquatic plants such as *Spirodela*) in combination with fluorescence measurements can also be used to monitor the presence of pesticides, heavy metals, and natural compounds that affect the photosynthetic apparatus. Snel et al. (1998) using a modulated PAM fluorometer and monitoring ETR followed the effect of low concentrations of linuron in microcosm experiments. Another example of the application of a PAM fluorometer was published by Perreault et al. (2010) who evaluated the effect of copper oxide nanoparticles on *Lemna gibba* using among other things the quenching analysis. Srivastava et al. (1998) using a PEA instrument showed that the cyanobacterial toxin fischerellin A caused an increase of *F*_J_; this indicates that fischerellin A affects the acceptor side of PSII like DCMU does. Bueno et al. (2004) showed an effect of lindane on the cyanobacterium *Anabaena*; they observed that this pesticide initially affects the amplitude of the JIP phase and after longer incubation times (12–24 h) causes a general suppression of the fluorescence intensity. In other studies, the effects of heavy metals like cadmium (Romanowska-Duda et al. 2005) or chromate (Susplugas et al. 2000) on *Spirodela oligorrhiza* have been studied. Finally, Chl *a* fluorescence is also a useful tool for the study of hydrogen production in e.g., *Chlamydomonas reinhardtii* (see e.g., Antal et al. 2006)

## Concluding remarks

For anyone who is beginning to use Chl *a* fluorescence, the overwhelming number of studies that already has been carried out may make it difficult to quickly discover what is already known and which experiments will add something new to the literature. Even so, it is important to formulate first some questions that are worth answering. Two points are worth keeping in mind. In the first place, the “flash,” “pulse,” and “steady state” communities live often in parallel universes; as a consequence, there are still many opportunities for a more integrated use of these techniques. In the second place, the currently available fluorescence devices can do much more than the few standard protocols that are most frequently used.

As this educational review suggests, there are many aspects of fluorescence that can be studied with different devices best adapted for the study of these different aspects. Flash experiments can be used to study the electron transfer reactions within PSII, direct fluorescence measurements are best for the measurement of the OJIP transients, which follow the reduction of the photosynthetic electron chain, and modulated measurements are best for steady state photosynthesis and the study of light-induced regulatory mechanisms affecting the antenna of PSII. The power of fluorescence techniques can be increased considerably by simultaneously measuring other parameters, such as 820 nm transmittance changes (probing PSI) or CO_2_ assimilation.

There are only a few basic principles that determine the yield of fluorescence. However, due to the fact that it is sensitive to many processes that differ between photosynthetic organisms, light acclimation states, intactness of samples, and stress conditions, a myriad of responses has been documented in the literature. The fluorescence literature may often be confusing and contradictory, but it contains a wealth of data and observations that we all need to understand. Only in that way, the wealth of information generated by past fluorescence research can be maximally exploited.

The contributing authors are available to be contacted by researchers for further discussions on the application of Chl *a* fluorescence through the following website: https://groups.google.com/forum/?hl=en#!forum/chlorophyllfluorescence where they will provide regular feedback.

## Abbreviations

*A*_n_: Net CO_2_ assimilation rate
ATP synthase: Enzyme responsible for the synthesis of ATP from ADP and inorganic phosphate
Car: Carotenoid
Chl: Chlorophyll
Chlz: Accessory chlorophyll in the photosystem II reaction center
CP43, CP47: Core antenna proteins of PSII of 43 and 47 kDa
Cyt b_6_/f: Cytochrome b_6_/f complex
D1 protein: One of the major PSII reaction center proteins, the other being D2
DBMIB: 2,5-Dibromo-3-methyl-6-isopropyl-p-benzoquinone
DCMU: 3-(3,4-Dichlorophenyl)-1,1-dimethylurea
DF: Delayed fluorescence
ETC: Electron transport chain
ETR: Electron transport rate
*F*_O_: Minimum Chl *a* fluorescence yield in the dark-adapted state
*F*_M_: Maximum Chl *a* fluorescence yield in the dark-adapted state
*F*_t_: Fluorescence intensity at time t
*F*_V_: Maximum variable fluorescence, defined as *F*_M_ − *F*_O_
*F*_V_/*F*_M_: A quantity related to the maximum quantum yield of PSII photochemistry
*F*_O_/*F*_M_: A parameter related to changes in heat dissipation in the photosystem II antenna
*F*_O_′, *F*_V_′, *F*_M_′, *F*_S_: Minimum, variable, maximum and steady state fluorescence intensity in the light-adapted state
*F*_q_′: *F*_M_′–*F*′ [with *F*′ = *F*s in the steady state]
*F*_q_′/*F*_M_′: Photosystem II operating efficiency
Fd: Ferredoxin
FER: Fluorescence excitation ratio
FNR: Ferredoxin-NADP^+^-reductase
*I*_1_: Fluorescence intensity at 2–3 ms
IRGA: Infra red gas analyzer
LED: Light-emitting diode
LHCII: Light harvesting complex II
NADP^+^: Nicotinamide adenine dinucleotide phosphate, oxidized form
NPQ: Non-photochemical quenching, expressed as (*F*_M_/*F*_M_′ − 1)
OJIP: transient Chl *a* fluorescence rise induced during a dark-to-strong light transition, where O is equivalent to *F*_O_, *P* is for peak, equivalent to *F*_M_ (when measured at saturating light) and J and I are inflections between O and P
O (*F*_O_), K (*F*_K_), J (*F*_J_), I (*F*_I_), P (*F*_P_): Fluorescence intensities at 20, 300 μs, 2–3 ms, ~30 and ~200 ms, respectively
P680: Photosystem II reaction center chlorophyll dimer
P700: Photosystem I reaction center chlorophyll dimer
PAM: Pulse amplitude modulation
PFD: Photon flux density
PEA: Photosynthesis efficiency analyser
PIabs: A JIP test parameter also called performance index
PQ: Plastoquinone
PSI, PSII: Photosystem I, Photosystem II
*Q*_A_: Primary quinone electron acceptors of PSII
*Q*_B_: Secondary quinone electron acceptor of PSII
qE, qT, qI: Non-photochemical quenching components defined by their relaxation times in darkness, where “E” stands for energy-dependent changes, “T” for state transitions, and “I” for photoinhibition
qN: Non-photochemical quenching, expressed as (1 − *F*_V_′/*F*_V_)
qP: Photochemical quenching
RLC: Rapid light curve
ROS: Reactive oxygen species
Rubisco: Ribulose-1,5-bisphosphate carboxylase/oxygenase
S-states S0 S1, S2, S3 and S4: Different redox states of the oxygen evolving complex
Sm: Normalized area above the OJIP transient
STF: Single turnover flash
TL: Thermoluminescence
XC: Xanthophyll cycle
UV: Ultraviolet
Δ*V*_IP_: Relative amplitude of the IP phase of Chl *a* fluorescence induction
*Φ*co_2_: Quantum yield of CO_2_ fixation
*Φ*_PSII_: PSII operating efficiency
